# Adsorption Site Regulations of [W–O]-Doped CoP Boosting the Hydrazine Oxidation-Coupled Hydrogen Evolution at Elevated Current Density

**DOI:** 10.1007/s40820-023-01185-4

**Published:** 2023-09-14

**Authors:** Ge Meng, Ziwei Chang, Libo Zhu, Chang Chen, Yafeng Chen, Han Tian, Wenshu Luo, Wenping Sun, Xiangzhi Cui, Jianlin Shi

**Affiliations:** 1 Shanghai Institute of Ceramics, Chinese Academy of Sciences, Shanghai, 200050 People’s Republic of China; 2 Center of Materials Science and Optoelectronics Engineering, University of Chinese Academy of Sciences, Beijing, 100049 People’s Republic of China; 3 State Key Laboratory of Clean Energy Utilization, School of Materials Science and Engineering, Zhejiang University, Hangzhou, 310027 People’s Republic of China; 4 School of Chemistry and Materials Science, Hangzhou Institute for Advanced Study, University of Chinese Academy of Sciences, Hangzhou, 310024 People’s Republic of China

**Keywords:** Self-powered H_2_ production system, Electron redistribution, [W–O] dopant, Dehydrogenation kinetics

## Abstract

Hydrazine oxidation reaction (HzOR) assisted hydrogen evolution reaction (HER) offers a feasible path for low power consumption to hydrogen production. Unfortunately however, the total electrooxidation of hydrazine in anode and the dissociation kinetics of water in cathode are critically depend on the interaction between the reaction intermediates and surface of catalysts, which are still challenging due to the totally different catalytic mechanisms. Herein, the [W–O] group with strong adsorption capacity is introduced into CoP nanoflakes to fabricate bifunctional catalyst, which possesses excellent catalytic performances towards both HER (185.60 mV at 1000 mA cm^−2^) and HzOR (78.99 mV at 10,00 mA cm^−2^) with the overall electrolyzer potential of 1.634 V lower than that of the water splitting system at 100 mA cm^−2^. The introduction of [W–O] groups, working as the adsorption sites for H_2_O dissociation and N_2_H_4_ dehydrogenation, leads to the formation of porous structure on CoP nanoflakes and regulates the electronic structure of Co through the linked O in [W–O] group as well, resultantly boosting the hydrogen production and HzOR. Moreover, a proof-of-concept direct hydrazine fuel cell-powered H_2_ production system has been assembled, realizing H_2_ evolution at a rate of 3.53 mmol cm^−2^ h^−1^ at room temperature without external electricity supply.

**Supplementary Information:**

The online version contains supplementary material available at 10.1007/s40820-023-01185-4.

## Highlights

- The [W–O] group with strong adsorption capacity is introduced into CoP to fabricate a bi-functional catalyst towards HER and HzOR.
- The cell voltage of HzOR coupled electrolyzer with 6W–O–CoP/NF as both anode and cathode catalysts is 1.634 V lower than that of the water splitting system at 100 mA cm^−2^.
- A proof-of-concept self-powered H_2_ production system is assembled to realize the H_2_ evolution rate of 3.53 mmol cm^−2^ h^−1^.

**Supplementary Information:**

The online version contains supplementary material available at 10.1007/s40820-023-01185-4.

## Graphical abstract

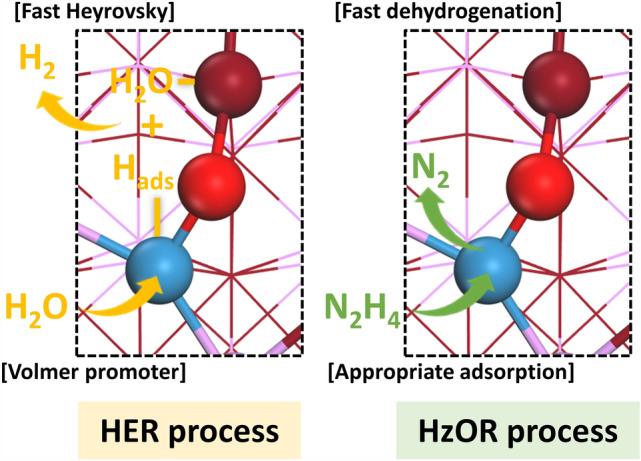

## Introduction

Substituting sluggish anodic oxygen evolution reaction (OER, 2OH^−^  → H_2_O + 1/2O_2_ + 2e^−^, 1.23 V vs. RHE) with thermodynamically favorable reactions, such as hydrazine oxidation reaction, (HzOR, N_2_H_4_ + 4OH^−^  → N_2_ + 4H_2_O + 4e^−^, − 0.33 V vs. RHE) to assist water electrolysis could greatly lower the electricity consumption for hydrogen production due to the ultra-low theoretical oxidation potential [1–4]. Developing bifunctional electrocatalysts toward anodic HzOR and cathodic HER in over hydrazine splitting system (OHzS) with high activity and durability is pivotal to promote the practical applications [5–9].On the one hand, as a four-electron process, the total electrooxidation of hydrazine is of great importance, not only for the efficient utilization N_2_H_4_, but also for preventing the potential generation of harmful NH_3_ originated from spontaneously decompose (3N_2_H_4_ → N_2_ + 2NH_3_) or incomplete oxidation (N_2_H_4_ + OH^−^  → 1/2N_2_ + NH_3_ + H_2_O + e^−^), which critically depends on the interaction between the reaction intermediates and surface of catalysts [10–12]. On the other hand, the catalytic performance of cathodic alkali hydrogen evolution is also tightly related with the surface characteristics of obtained catalysts [13–16]. Thus, designing efficient and low-cost bifunctional catalysts with moderate adsorption energy and ΔG_H*_ is of great significance to achieve low energy consumption and high efficiency of green hydrogen production in OHzS.

Recently, 3d transition metal phosphides, such as CoP, have shown excellent catalytic activity toward HER and HzOR, which are regarded as the potential alternatives to Pt-based catalysts and applied in OHzS [17–22]. Unfortunately, the performance of the reported CoP-based catalysts is far from practical application mainly due to the low adsorption ability in alkaline electrolyte, which subsequently inhibits the following water dissociation reaction (Volmer step) and total hydrazine electrooxidation via 4-electron process resulting in the decay of catalytic activity [23, 24]. To address the above issue, two strategies have been proposed. First approach is introducing adsorption promoter, such as metal oxides to fabricate CoP/oxide hybrid [25–28]. However, the limited active site density and the additional charge transfer resistance result in the undesirable catalytic performance [29]. Second approach is anion doping, especially electronegative oxygen-incorporation, to tailor the 3d electronic structure and thus optimize the intrinsic adsorption ability [30–32]. Nevertheless, an ideal balance between the high active site exposure and optimal ad-desorption energy of CoP-based catalysts cannot be obtained based on the above adsorption promoter and oxygen-incorporation strategies [32]. More importantly, as a relatively early electrocatalysis system, the underlying HzOR mechanism is still unclear theoretically.

In this work, the tungsten bridged oxygen [W–O] species of strong adsorption capacity were introduced into CoP nanoflakes (denoted as 6W–O–CoP/NF) via an in situ hydrolysis etching method. The introduced [W–O] species can not only work as the strong adsorption sites toward both H_2_O and N_2_H_4_ molecules, respectively accelerating the cathodic H_2_O dissociation and the anodic complete N_2_H_4_ oxidation, but also regulate the *d*-band center of Co sites through the bridged O in [W–O] group to subsequently facilitate the Heyrovsky step, finally leading to the high enhanced catalytic performances. Moreover, the introduction of [W–O] simultaneously induces the porous structure in CoP nanoflakes, resulting in the high exposed active sites, excellent hydrophilicity as well as the fast mass transfer kinetics. The porous W–O-CoP hybrid nanoflakes endow the obtained 6W–O–CoP/NF industry-level catalytic activities toward both HER and HzOR, especially an overpotential of 78.99 mV@1000 mA cm^−2^ and an ultra-low Tafel slope of 8.43 mV dec^−1^ are obtained for HzOR. Both the in situ Raman and RDE tests exhibit the potential HzOR determining step (PDS) for W–O–CoP hybrid catalyst (^*^N_2_H_2_ → ^*^N_2_H) could be easily overcame within the overpotential of 0 V (vs. RHE), revealing the fast kinetics and 4-electron process toward HzOR. Benefiting from the high catalytic activity and excellent hydrophilicity structure of the W–O–CoP/NF electrode, the equipped OHzS possesses an ultra-low operation voltage of 0.165 V to reach 100 mA cm^−2^, 1.634 V lower than that of over water splitting (OWS). A proof-of-concept self-powered H_2_ production system has been assembled, realizing a decent H_2_ evolution rate of 3.53 mmol cm^-^^2^ h at room temperature without external electricity supply.

## Experimental Section

### Materials

Cobalt nitrate hexahydrate (Co(NO)_3_·6H_2_O) and Hydrochloric acid (HCl) were purchased from Reagent. 2-Methylimidazole (C_4_H_6_N_2_) was obtained from Adamas. Sodium tungsten dihydrate (Na_2_WO_4_) was purchased from Sigma-Aldrich. Sodium hypophosphite (NaH_2_PO_4_) was purchased from Shanghai Aladdin. Ethanol (C_2_H_5_OH) was obtained from Shanghai Lingfeng. Commercial 20 wt% Pt/C was purchased from Shanghai HEPHAS Energy Equipment.

### Preparation of Catalysts

#### Pre-treatment of Nickle Foam

Ni foam ( 2 × 2 cm^2^) was cleaned in 3 M HCl, ethanol and deionized water with ultrasonication for 15 min, respectively. And then dried in a vacuum oven at 60 °C for 12 h to obtain fresh NF.

#### Preparation of ZIF-L Nanoarrays on NF

An aqueous solution containing 0.4 M 2-methylimidazole (C_4_H_6_N_2_, 40 mL) was quickly poured into the solution of 40 mL 0.05 M Co(NO_3_)_2_·6H_2_O. After vigorous stirring for 10 min, the piece of NF substrate was immersed into the mixture solution to react for 4 h (200 rpm) and then was taken out, cleaned with deionized water and dried in a vacuum oven at 60 °C for 12 h to obtain ZIF-L/NF.

#### Preparation of W–O-Co MOF Nanoarrays on NF

A piece of ZIF-L/NF was immersed into the mixture solution of 100 mL ethanol/deionized water (1:4 in volume) containing different amount of Na_2_WO_4_ (0, 0.2, 0.4, 0.6, 0.8, and 1.0 g). After reacting at 85 °C for 15 min (300 rpm), the color of solution changed from purple into translucent. Then, the sample was taken out quickly, washed with deionized water three times and then dried in a vacuum oven at 60 °C for 12 h to obtained W–O–Co MOF/NF (denoted as 0-W–O–Co MOF/NF, 2-W–O–Co MOF/NF, 4-W–O–Co MOF/NF, 6-W–O–Co MOF/NF, 8-W–O–Co MOF/NF, 10-W–O–Co MOF/NF, respectively).

#### Preparation of W–O-CoP Nanoarrays on NF

A piece of W–O–Co MOF/NF and 500 mg NaH_2_PO_4_ powders were placed at two ceramic boat, and NaH_2_PO_4_ powders was placed at the upstream side of tube furnace. Then, the W–O–Co MOF/NF was annealed and phosphatized at 350 °C for 2 h with a heating rate of 2 °C min^−1^ under nitrogen atmosphere and then the W–O–CoP/NF was obtained.

#### Preparation of CoP Nanoarrays on NF

CoP/NF was obtained by directly phosphating the pre-synthesized ZIF-L/NF under the same phosphating conditions without the addition of Na_2_WO_4_.

#### Preparation of P-NF Nanoarrays on NF

P-NF was obtained by directly phosphating the pre-treated NF under the same phosphating condition.

### Characterization of Catalysts

The scanning electron microscope (SEM) images were carried out on a FEI Magellan-400 field emission scanning electron microscope (5 kV) and a Hitachi S-4800 field emission scanning electron microscope (3 kV). The transmission electron microscope (TEM) and high-resolution TEM (HRTEM) patterns were performed by a JEM-2100F field emission transmission electron microscope (200 kV). The X-ray diffraction (XRD) patterns were recorded using a Rigaku D/Max-2550 V X-ray diffractometer with a Cu Kα radiation target (40 kV, 40 mA). The X-ray photoelectron spectroscope (XPS) signals were measured on a Thermo Fisher Scientific ECSAlab250 XPS spectrometer with monochromatic Mg Kα X-rays and were calibrated with carbon base (284.8 eV). Fourier transform infrared (FTIR) spectroscopy was carried out on a Nicolet iS10 FTIR spectrometer using the KBr technique. The Raman spectra were measured on a GX-PT-1500 (150) instrument with an excitation wavelength of 532 nm. The electrochemical in situ Raman spectrometer cell (EC-RAIR-H) with graphite rod and Ag/AgCl as counter and reference electrodes, respectively, was purchased from Beijing Scistar Technology Co. Ltd.

### Electrochemical Measurements

Electrochemical performances of the samples were performed in N_2_-saturated 1 M KOH solution or 1 M KOH + 0.1 M N_2_H_4_ solution by using an electrochemical workstation (CHI Instruments 760E, China). In a typical three-electrode system, the as-prepared sample was applied as work electrode directly with Ag/AgCl as reference electrode and graphite rod as counter electrode. The mass loading amount of 6W-O-CoP and commercial Pt/C on Ni Foam is ~ 2.6 and ~ 1 mg cm^−2^, respectively. All potential data reported in this work were calibrated relatively to the reversible hydrogen electrode (RHE) scale according to the Nernst equation (*E*_RHE_ = *E*_Ag/AgCl_ + 0.0592 × pH + 0.1989 V), in which *E*_Ag/AgCl_ is the external potential measured against the Ag/AgCl reference electrode. Liner sweep voltammetry (LSV) curves were carried out at a scan rate of 5 mV s^−1^ with 98% iR compensations. The Tafel slope was calculated by fitting the liner part of the Tafel plots according to the Tafel equation (*η* = *a* + *b* log(*j*)). The electrochemical impedance measurements were performed in a frequency range of 0.01 Hz to 10^5^ Hz with an amplitude of 5 mV. The double layer capacitance (*C*_dl_) of the obtained samples was calculated from CV curves at different scan rates of 20–100 mV s^−1^ in the non-Faraday area. Faradic efficiency (FE) was measured by the gas chromatograph (GC) with a thermal conductivity detector according to the following equation:

$${\text{FE}} = nzF/Q*100\%$$

where *n* is the measured amount of H_2_ gas (mol), *z* is the electron transfer number, *F* is the Faraday constant (96,485 C mol^-1^), and *Q* is the total charge which can be obtained through the electrochemical workstation.

The HzOR-assisted OWS experiments were performed in a H-type two electrodes cell, and the AEM (anion exchange membrane) was used to divide anode and cathode and allow anions to transfer. The as-prepared samples were used as both anode and cathode and filled with 1 M KOH + 0.1 M N_2_H_4_ or 1 M KOH, respectively.

### Direct Hydrazine Fuel Cell (DHzFC) Preparation and Measurements

The as-prepared 6W–O–CoP/NF (0.24 cm^2^) was investigated as anode catalyst layer in membrane electrode assembly (MEA) testing. 0.5 mg cm^−2^ loading of commercial 20 wt% Pt/C on composite substrate was used as the cathode layer. The DHzFC device was evaluated using a CHI 760E electrochemistry workstation at room temperature, with 1 M KOH + 0.1 M N_2_H_4_ as anode electrolyte and 1 M KOH as cathode electrolyte with air passing into the cathode side.

### Computational Method

All the theoretical calculations were carried out with density functional theory (DFT) method as implemented in the Vienna Ab Initio Simulation Package (VASP) [33]. The electron ion interaction was described with the projector augmented wave (PAW) method [34] while the electron exchange and correlation energy were solved within the generalized gradient approximation with the revised Perdew–Burke–Ernzerhof (RPBE) exchange–correlation functional [35, 36]. The empirical correction in Grimme’s method (DFT + D3) was used to describe van der Waals interaction [37] and the dipole correction was employed to correct potential spurious terms arising from the asymmetry of the slabs [38]. The kinetic energy cutoff of plane wave was set to be 400 eV, and the convergence criterion for the residual forces and total energies were set to be 0.03 eV Å^−1^ and 10^−5^ eV, respectively. The pure CoP (011) surface consists of four atomic layers and 3 × 1 supercells, while the W–O-doped catalyst is constructed by replacing surface one Co and one P on the surface with W and O atoms. The bottom two layers were fixed during the structural relaxation. A vacuum layer of 20 Å along the z direction was set between the periodically repeated slabs to avoid strong interactions, and a 3 × 3 × 1 Monkhorst–Pack k-point grids was used to sample the Brillouin zone. Transition state with only one imaginary frequency was conducted with the climbing image nudged elastic band (CI-NEB) method [39]. Bader charge calculation was performed to analyze the charge population and charge transfer [40].

The adsorption energy (*E*_ads_) was calculated based on the equation:

$$E_{{{\text{ads}}}} = E_{{{\text{total}}}} {-}E_{{{\text{substrate}}}} {-}E_{{{\text{adsorbate}}}}$$

where *E*_total_, *E*_substrate_ and *E*_adsorbate_ represent the total energies of the systems containing the substrate and adsorbate, the substrate, and the adsorbate, respectively. According to this definition, a more negative adsorption energy indicates a stronger interaction.

The Gibbs free energy change (Δ*G*) of each elementary step was calculated by using the computational hydrogen electrode (CHE) model proposed by Nørskov et al. [41]. In this model, the chemical potential of the proton–electron pair in aqueous solution is related to that of one-half of the chemical potential of an isolated hydrogen molecule. The Δ*G* value can be obtained by the formula: Δ*G* = Δ*E* + Δ*ZPE* − *T*Δ*S*, where Δ*E* is the reaction energy of reactant and product species adsorbed on the catalyst directly obtained from DFT calculations; Δ*ZPE* and Δ*S* are the changes between the adsorbed species and the gas phase molecules in zero-point energies and entropy at 298.15 K, which can be calculated from the vibrational frequencies.

## Results and Discussion

### Theoretical Investigations

**Fig. 1:**
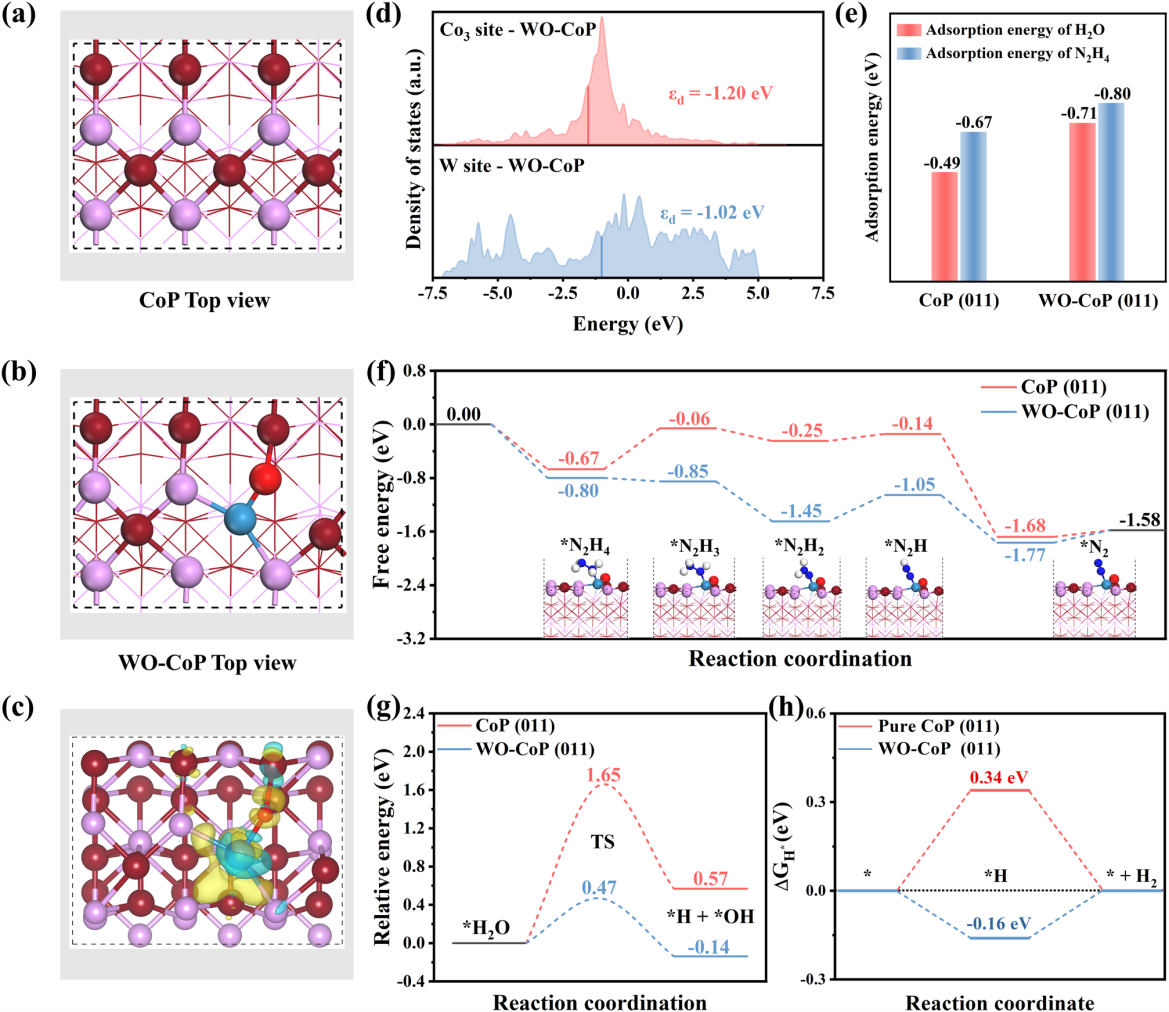
Top views of **a** CoP (011) structure model and** b** WO–CoP (011) structure model (Co-brownish red, P-pink, W-blue, O-red). **c** The calculated charge density difference of WO–CoP. **d** Density of states (DOS) of Co_3_ site and W site in WO–CoP. **e** Adsorption energy on pure CoP and WO–CoP. **f** The free energy diagrams of hydrazine dehydrogenation, **g** the free energy diagrams of water dissociation and **h** the Gibbs free energy diagrams of pure CoP and WO–CoP. (Color figure online)

To design the bifunctional catalyst, the structure sensitive of catalyst toward both HzOR and HER is considered, which is highly related to the interaction between absorbates and catalyst’s surface [10, 42, 43]. In fact, the weak adsorption ability on CoP hinders the N_2_H_4_ dehydrogenation as well as H_2_O dissociation [30]. While the high-valence W-based species possess more outermost vacant orbitals (*d*° system), which could behave as the adsorption site for reactants, and also regulate the electronic structure of CoP substrate to optimize its intrinsic catalytic activity [27, 35–46]. Based on this, before carrying out the experiment, the first principle calculation was adopted to reveal the effect of [W–O] species introduction on CoP by replacing one Co and one P atoms on the surface of CoP (011) by W and O atoms, respectively, as illustrated in Fig. 1a, b.

Firstly, the charge density difference of WO–CoP hybrid was calculated to reveal the electron distribution between the doped W–O and CoP substrate. As shown in Fig. 1c, the [W–O] hybrid would induce the charge depletion around Co and W (green region) and the charge accumulation around P and O atoms (orange region), where the formation of positive centers (Co or W sites) favors the preferential adsorption of H_2_O and N_2_H_4_ molecules [47]. In order to further clarify the possible adsorption site in [W–O]-doped CoP, we further calculated the *d*-band centers of each Co and W sites before and after [W–O] replacement as illustrated in Figs. 1d and S1. It’s worth noting that the *d*-band center of Co atoms bridged [W–O] site (Co_3_ site in Fig. S1a) notably shifts from − 1.36 to − 1.20 eV after [W–O] substitution in Co_5_ site, demonstrating the elevated bind with the reaction intermediate. Furthermore, compared with Co_3_ site (− 1.20 eV) in WO–CoP, the *d*-band center of substituted [W–O] is much closer to the Fermi level (− 1.02 eV, Fig. 1d), indicating that the [W–O] dopant could preferentially absorb the H_2_O and N_2_H_4_ molecules according to the *d*-band center theory and then improve the following water dissociation as well as hydrazine oxidation kinetics to simultaneously boost the alkaline HER and HzOR [27, 48, 49]. Furthermore, the water adsorption energy ($\Delta {\text{E}}_{{{\text{H}}_{2} {\text{O}}}}$) as well as the hydrazine adsorption energy ($\Delta {\text{E}}_{{{\text{N}}_{2} {\text{H}}_{4} }}$) on WO–CoP surface is also carried out in Fig. 1e, exhibiting the enhanced adsorption process with spontaneity in comparison with pure CoP (011), verifying the optimized interaction between absorbates and catalyst’s surface and thus guaranteeing the ongoing hydrazine dehydrogenation and water dissociation on the introduced [W–O] sites.

The corresponding catalytic barriers of WO–CoP and CoP were then analyzed and are illustrated in Fig. 1f–h. For anodic HzOR, the complete oxidation of N_2_H_4_ is a four-continuous-step proton-coupled electron transfer (PCET) process [50]. Thus, the free energies of each dehydrogenation step from adsorbed ^*^N_2_H_4_ to N_2_ (^*^N_2_H_4_ → ^*^N_2_H_3_ → ^*^N_2_H_2_ → ^*^N_2_H → ^*^N_2_ → N_2_) on [W–O] site of WO–CoP were calculated (Figs. 1f and S2). The potential-determining step (PDS) for WO–CoP is the dehydrogenation from ^*^N_2_H_2_ to ^*^N_2_H. As expected, a much lower energy barrier (Δ*G*) value of + 0.40 eV is obtained of WO–CoP in comparison with the pure CoP (+ 0.61 eV), indicating the facilitation of dehydrogenation kinetics in WO–CoP toward HzOR. For cathodic HER, the calculated water dissociation barrier (Figs. 1g and S3) on the [W–O] site (0.47 eV) is much lower than that of pure CoP (1.65 eV), indicating the accelerated Volmer step (H_2_O + e^−^  + * → H_ads_ + OH^−^) and more H_ads_ species produced on [W–O] sites. Then, the absorbed H_2_O on Co_3_ site linked with [W–O] could combine with the generated H_ads_ species on adjacent [W–O] site to co-produce H_2_ (H_ads_ + H_2_O + e^−^  → H_2_ + OH^−^). Meanwhile, the WO–CoP hybrid also possesses the relative moderate Δ*G*_H*_ value (− 0.16 eV), implying the favorable hydrogen ad-desorption thermodynamics of [W–O]-doped CoP (Figs. 1h and S4). The results consolidate that the introduced [W–O] group not only work as the potential active sites for both HER and HzOR, but also effectively optimize the electronic structure of CoP substrate, which could achieve an ideal balance between the high active site exposure and optimal ad-desorption energy among CoP-based catalysts.

### Synthesis and Characterization

**Fig. 2:**
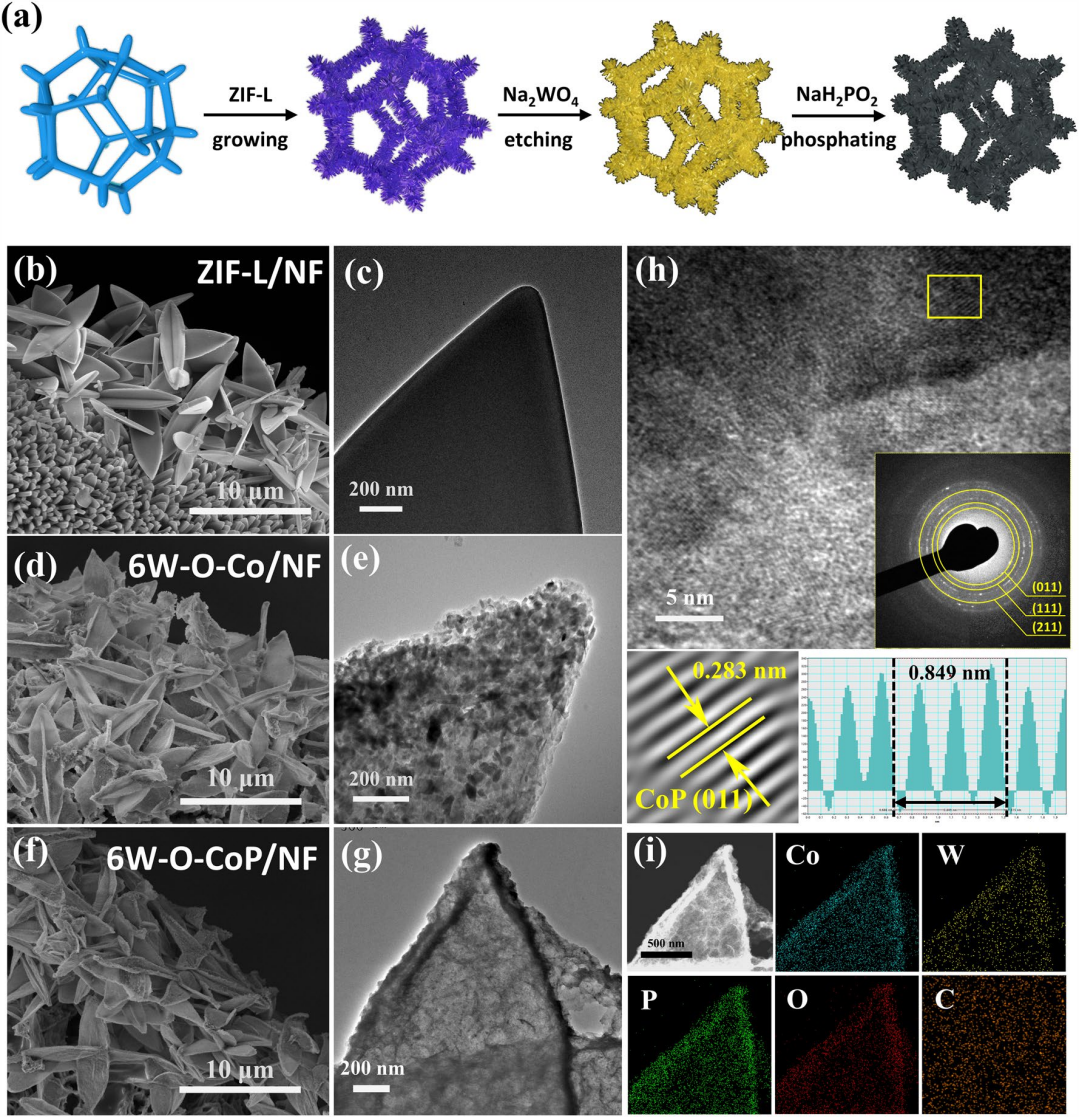
**a** Schematics for the synthesis of hollow and porous W–O–CoP/NF nanoflakes. **b** SEM and **c** TEM images of ZIF-L precursor.** d** SEM and **e** TEM images of 6W–O–Co/NF. **f** SEM, **g** TEM, **h** HRTEM (inset: SAED pattern) and **i** the corresponding EDS mapping images of 6W–O–CoP/NF

As suggested by the DFT calculation results, the hierarchical W–O–CoP nanoarrays on Ni foam (NF) have been fabricated via an in situ hydrolysis etching/doping route (Fig. 2a). Firstly, the nanosheet-like ZIF-L was directly grown on NF through a facile reaction between Co^2+^ and 2-MIM. Then, the precursor ZIF-L was in situ etched into a porous structure in the mixture of ethanol and deionized water containing Na_2_WO_4_, in which the hydrolysis of water could produce H^+^ and OH^−^ to etch ZIF-L, in situ dope [WO_4_]^2−^ and accelerate the deposition of Co^2+^ simultaneously. After phosphating treatment, the highly porous CoP-doped [W–O] species was obtained (denoted as xW–O–CoP/NF, x refers to the addition amount of Na_2_WO_4_ precursor).

The morphology and structure of the pre-synthesized ZIF-L on NF (denoted as ZIF-L/NF) were characterized by an electron microscopic technique (Figs. 2b, c and S5), which show the vertically aligned leaf-like morphology possessing an ultra-smooth surface and a uniform size distribution with the width of ~ 3 μm. The XRD pattern of ZIF-L also exhibits the same two-dimensional layered crystal structure as the reported zinc analogue (Fig. S6) [51]. During the hydrolysis reaction, ZIF-L was in situ etched and doped, in which the [WO_4_]^2−^ cluster was concurrently introduced, leading to the formation of the porously leaf-like nanoflakes (denoted as 6W-O-Co/NF) (Figs. 2d, e and S7). The smooth surface becomes “semitransparent” in obtained 6W-O-Co/NF with numerous ultra-small scale-flakes grown on. The phase of 6W-O-Co is well indexed as Co-LDH (JCPDS#50–0235) (Fig. S8). The existence of introduced [WO_4_]^2−^ species could be detected by the Raman spectra (Fig. S9), in which the characteristic peaks appeared at around 800 ~ 950 cm^−1^, confirming the successful introduction of [WO_4_]^2−^ clusters in Co-LDH [52, 53]. After phosphation, the 6W–O–CoP/NF catalyst was obtained, still retaining the semitransparent and curled leaf-like nanoflakes with rough surface aligned on NF (Figs. 2f, g and S10). HRTEM demonstrates the interplanar distance of 0.283 nm, corresponding to the (011) plane of CoP in 6W–O–CoP/NF (Fig. 2h), verifying the successful synthesis of orthorhombic CoP. The uniform distributions of Co, P, W and O elements in 6W–O–CoP/NF nanoflakes further confirm the successful doping of [W–O] species in CoP substrate without aggregations (Figs. 2i and S11).

**Fig. 3:**
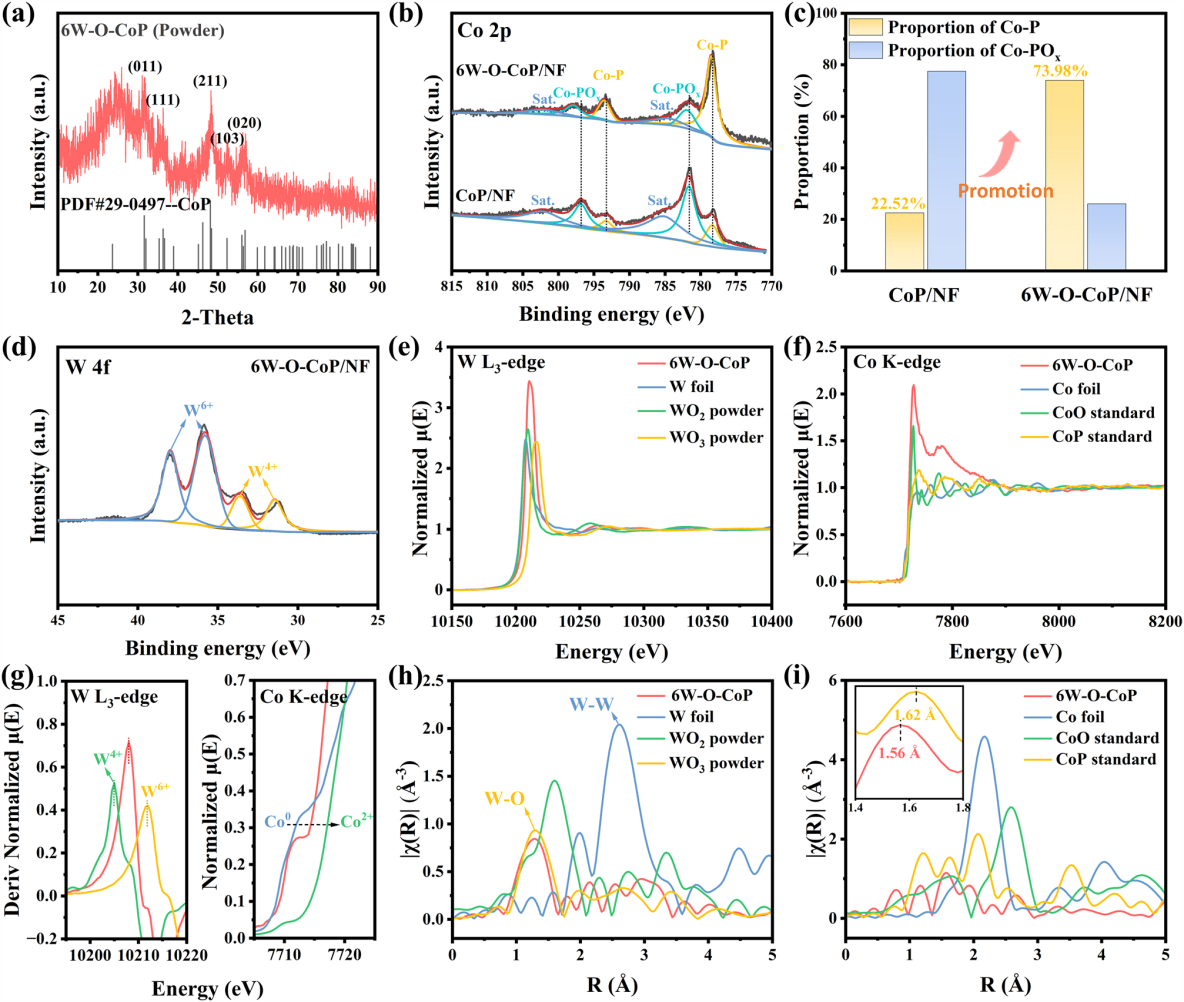
**a** XRD patterns of 6W–O–CoP powders sonicated from NF support. **b** High-resolution XPS spectra of Co 2*p* and **c** the corresponding proportion histograms of Co-P and Co-PO_x_ in prepared catalysts. **d** High-resolution XPS spectra of W 4*f*. **e** W L_3_-edge and **f** Co K-edge XANES spectra of 6W–O–CoP/NF and the reference compounds. **g** The first derivation curves of W L_3_-edge (left) and the partial enlargements of Co L-edge (right). EXAFS spectra of **h** W L_3_-edge and **i** Co K-edge in the R-space of 6W-O-CoP and the references

From the XRD pattern in Fig. 3a, the diffraction peaks of obtained 6W-O-CoP sonicated from the NF are well in line with the standard CoP (JCPDS#29–0497) with no characteristic diffraction peaks of tungsten-based species found, indicating the negligible effect of [WO_4_]^2−^ introduction on the crystalline phase [54, 55]. Raman spectra of 6W–O–CoP/NF in Fig. S12 exhibit two characteristic peaks at 1338 and 1590 cm^−1^, being ascribed to the disorder/defective carbon and *sp*^2^ hybridized graphitic carbon, respectively. Noteworthily, the *I*_D_/*I*_G_ ratio of 6W–O–CoP/NF increases from 0.84 to 1.00 in comparison with the prepared CoP/NF without etching/doping, meaning that the etching/doping process could break the original structure of ZIF-L and then lead to the extrinsic disorders increase [56, 57]. The characteristic peak position at 880 cm^−1^ in 6W–O–CoP/NF is well related to the stretching mode of W–O bond (Fig. S12) [58], verifying the successful introduction of [W–O] species in CoP nanoflakes. According to the ICP-OES result, the W doping amount in 6W–O–CoP/NF catalyst is 1.03 wt% (Table S1).

XPS measurement was performed to gain the detailed information on the surface composition and binding structure of obtained catalysts. Figure S13a depicts the survey spectrum of 6W–O–CoP/NF, evidencing the co-existence of Co, P, W and O elements, in consistent with the EDS result in Figs. 2i and S11. Co 2*p* spectra (Fig. 3b) of 6W–O–CoP/NF exhibits six subpeaks at 778.52 and 793.60 eV, 781.88 and 797.90 eV, 784.50 and 802.00 eV, respectively corresponding to Co-P bonds, Co-PO_x_ bonds and satellite peaks [59, 60]. Notably, compared with CoP/NF, the etching/doping process could elevate the concentration of Co–P bonds from 22.52% to 73.98%, indicating the enhanced phosphation of Co species to form CoP phase by hydrolysis and doping process (Fig. 3c) [61, 62]. Furthermore, the binding energies of 6W–O–CoP/NF are up-shifted by ~ 0.34 eV compared with CoP/NF (778.30 and 793.26 eV, 781.60 and 796.78 eV), demonstrating the modified electronic structure of Co resulting from the [W–O] doping, agreeing well with the DFT results (Fig. 1) [63]. Figure 3d shows the high resolution of W 4*f* spectra with four peaks, in which the bands at 31.44 and 33.62 eV are attributed to W^4+^, while the others at 35.79 and 37.97 eV are indexed to W^6+^, implying the co-existence of high valence tungsten [64]. P 2*p* spectrum of 6W–O–CoP/NF exhibits three peaks at 129.52, 130.44 and 133.66 eV, ascribed to P 2*p*_3/2_ and P 2*p*_1/2_ of P–Co bonds and P–O bonds, respectively (Fig. S14). Meanwhile, the O 1* s* spectra of 6W–O–CoP/NF in Fig. S15 show three peaks at about 530.54 eV, 531.46 eV and 533.31 eV, representing metal-O, absorbed H_2_O and P–O, respectively [65, 66]. It worth noting that the O 1*s* spectra of 6W–O–CoP/NF show the relatively negative shifts compared with CoP/NF, implying the electrons accumulated in doped electronegative O atoms, leading to the optimized electron distribution of obtained hybrid W–O–CoP/NF.

To further investigate the fine chemical state and coordination environment of 6W–O–CoP/NF catalyst, both the XANES and the EXAFS of W L_3_-edge and Co K-edge were conducted and are shown in Fig. 3e–i, respectively. The W L_3_-edge threshold *E*_0_ of obtained 6W–O–CoP is located in between those of WO_2_ and WO_3_ powder (Fig. 3g-left), implying that the average valance state of W is in between + 4 and + 6, in agreement with the high-resolution XPS result of W 4f (Fig. 3d) [67]. Meanwhile, the Co K-edge XANES spectra of 6W–O–CoP are located in between those of Co foil and standard CoO (Fig. 3g-right), indicating the average valence state of Co in between 0 and + 2 [68]. More detailed local chemical coordination of doped W atoms in 6W–O–CoP is manifested in Fig. 3h. The peak located at around 1.3 Å is originated from the W–O bonding, indicating that the doped W atom in 6W–O–CoP is mainly bonded with O atoms in accordance with the Raman spectra in Fig. S12, consolidating the successful introduction of [W–O] into the CoP structure. In addition, no apparent peaks for W-W bonds (2.00 and 2.61 Å) can be detected, confirming the homogenously disperse of doped [W–O] group with no particle aggregations [69]. Figure 3i shows the local chemical environment of Co, in which the atomic distance of 6W-O-CoP (1.56 Å) slightly downshifts in comparison with standard CoP (1.62 Å) due to the in situ formation of shorter Co–O bond after the [W–O] introduction [70]. The formation of Co–O bond evidences the strengthened interaction between CoP substrate and doped [W–O] group, verifying the successful hybrid of [W–O] with CoP resultantly facilitating the charge transfer in the electrocatalytic processes.

### Electrocatalytic Performances

**Fig. 4:**
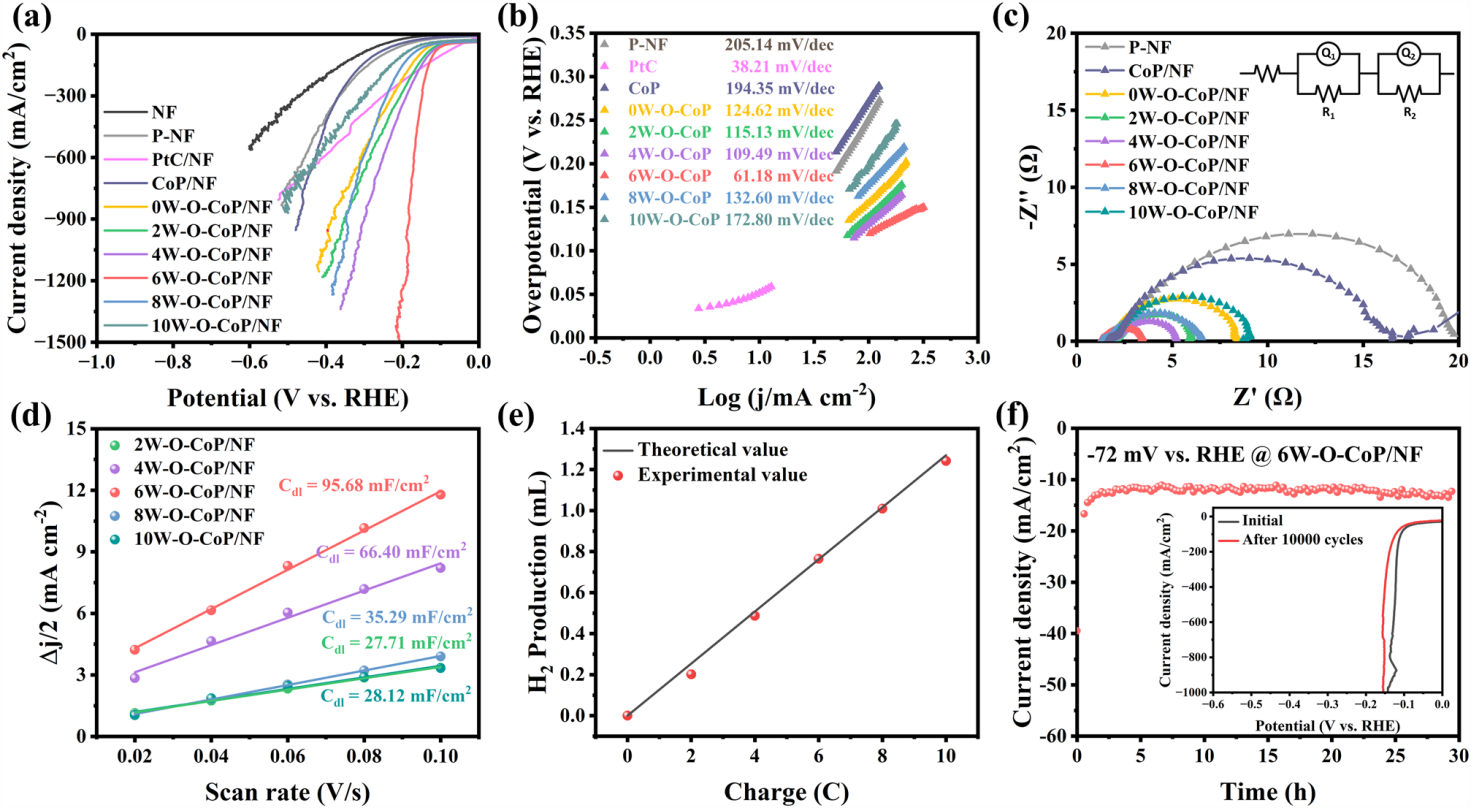
HER performance in 1.0 M KOH. **a** HER polarization curves of NF, P-NF, commercial PtC/NF, CoP/NF and xW–O–CoP/NF-based catalysts (*x* = 0, 2, 4, 6, 8, 10) and **b** the corresponding Tafel slopes. **c** Nyquist plots of the catalysts with the equivalent circuit in the insets. **d** Plots of current density (Δ*j*/2) versus scan rate for determining *C*_dl_ values. **e** Comparison between GC-measured and theoretically calculated H_2_ quantities of 6W–O–CoP/NF. **f** Stability measurement for 6W–O–CoP/NF at overpotential of − 72 mV for 30 h (inset: the LSV curves before and after 10,000 HER cycles)

The HER performance of obtained catalysts was investigated in 1.0 M KOH electrolyte at a scan rate of 5 mV s^−1^. Figure 4a exhibits the polarization curves of the obtained catalysts after 98% iR-correction. xW–O–CoP/NF-based catalysts with varied [WO_4_]^2−^ addition amounts were conducted. Compared with bare NF and P-NF, NF with CoP nanoflakes grown on exhibits better HER catalytic performance, indicating the intrinsic HER catalytic activity of CoP [71]. After the hydrolysis etching and [W–O] doping, the xW–O–CoP/NF-based catalysts possess the much elevated HER catalytic performance (Fig. 4a), and the 6W–O–CoP/NF with the optimal addition amount of [WO_4_]^2−^ presents the highest HER catalytic activity, featuring the markedly low overpotentials of 84.1, 120.0, 164.4, 185.6 and 202.2 mV to reach the current densities of 50, 100, 500, 1000 and 1500 mA cm^−2^, respectively. Impressively, 6W–O–CoP/NF demonstrates much higher HER activity than the ever-reported self-supported transition metal-based catalysts, especially at large current density (> 1000 mA cm^−2^, Table S2). This is not only closely connected with the modified adsorption sites of [W–O] group and the optimized electronic structure of CoP substrate, but also related with the extremely hydrophilic surface of porous xW–O–CoP/NF nanoflakes (Fig. S16), which is more favorable for the accessibility of electrolyte and release of produced H_2_. Moreover, the xW–O–CoP/NF-based catalysts exhibit the relative lower Tafel slopes than that of CoP/NF (194.35 mV dec^−1^) (Fig. 4b), illustrating that the [W–O] doping in CoP are responsible for the strengthened H_2_O adsorption and subsequent dissociation kinetics toward HER (Volmer step: H_2_O + e^−^  + * → H_ads_ + OH^−^) [72]. Moreover, the exchange current density (*j*_0_) extrapolated from the Tafel plots of 6W–O–CoP/NF is 1.077 mA cm^−2^, demonstrating the favorable intrinsic electrocatalytic activity.

The 6W–O–CoP/NF electrode exhibits the lowest charge transfer resistance (*R*_ct_) of 1.40 Ω compared to other samples (CoP: *R*_ct_ = 13.82 Ω; 0W: *R*_ct_ = 6.29 Ω; 2W: *R*_ct_ = 3.26 Ω; 4W: *R*_ct_ = 2.54 Ω; 8W: *R*_ct_ = 4.64 Ω; 10W: *R*_ct_ = 6.51 Ω) according to the electrochemical impedance spectroscopy (EIS) measurements and fitted data (Fig. 4c and Table S3), meaning the fast electron transfer of 6W–O–CoP/NF catalyst during HER process. Furthermore, the electrochemical double layer capacitance (*C*_dl_) has been calculated as shown in Figs. 4d and S17. The 6W–O–CoP/NF exhibits the highest *C*_dl_ value of 95.68 mF cm^−2^, meaning the highest catalytically active surface area [73]. The hydrogen production faradic efficiency was measured by the gas chromatograph (GC, Fig. 4e), and an average Faradic efficiency of nearly 100% was obtained on 6W–O–CoP/NF. Both the long-term durability test with high current density retention after 30 h and the polarization curves before and after 10,000 CV test nearly overlapped with each other indicate the excellent HER stability of 6W–O–CoP/NF (Fig. 4f). Meanwhile, the similar morphology and phase of 6W–O–CoP/NF after 10,000 cycles with those of the initial (Figs. S18–S20) indicates the excellent structure stability of 6W–O–CoP/NF during HER. Furthermore, the long-term durability test of 6W–O–CoP/NF at high current density was carried out, which also exhibits an excellent HER stability (Fig. S21) and an average Faradic efficiency of up to 100% (Fig. S22).

**Fig. 5:**
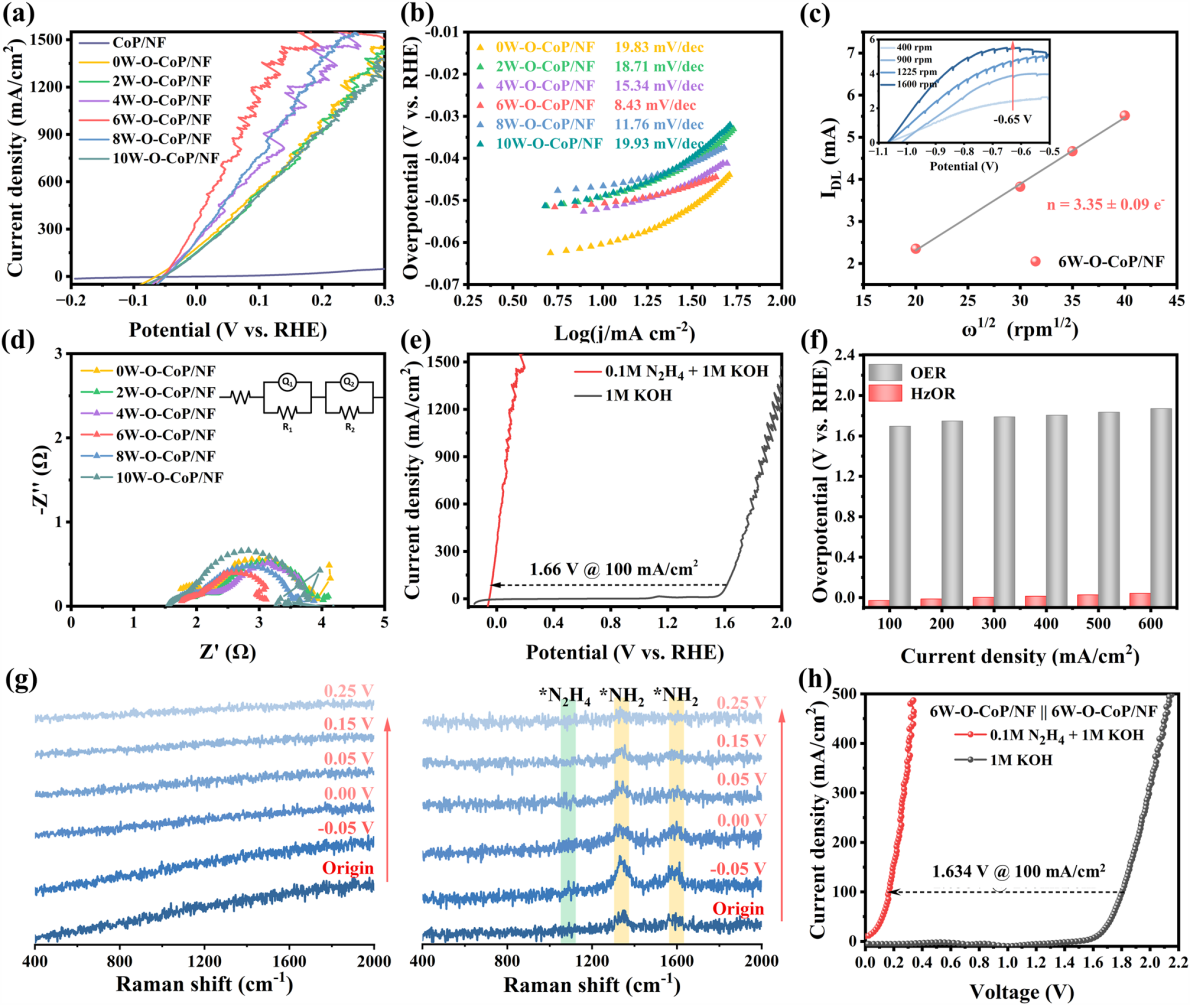
HzOR performance in 1.0 M KOH + 0.1 M N_2_H_4_ electrolyte. **a** Polarization curves of CoP/NF and xW–O–CoP/NF-based catalysts (*x* = 0, 2, 4, 6, 8, 10) and the corresponding **b** Tafel slopes. **c** Liner fitting of the diffusion limited current at different rotation (inset: LSV curves at different rotation). **d** Nyquist plots with the equivalent circuit in the insets. **e** Compared LSV curves of 6W–O–CoP/NF between OER (1.0 M KOH) and HzOR (1.0 M KOH + 0.1 M N_2_H_4_), and** f** the corresponding histogram catalytic performance comparison. **g** The in situ electrochemical Raman spectra of 6W–O–CoP/NF in 1.0 M KOH (left) and 1.0 M KOH + 0.1 M N_2_H_4_ (right) at varied applied potentials. **h** Comparison LSV curves of 6W–O–CoP/NF between OWS and OHzS

The HzOR electrochemical performance of xW–O–CoP/NF-based catalysts was then evaluated in 1.0 M KOH + 0.1 M N_2_H_4_ electrolyte. From Fig. 5a, 6W–O–CoP/NF still exhibits the best HzOR catalytic activity among the prepared samples with overpotentials of only − 50.65, − 34.74 and 78.99 mV to reach 10, 100 and 1000 mA cm^−2^, respectively. A small Tafel slope of 8.43 mV dec^−1^ was obtained in 6W–O–CoP/NF, indicating the favorable catalytic oxidation kinetics toward HzOR (Fig. 5b). Compared with the reported HzOR catalysts (Table S4), the prepared 6W–O–CoP/NF catalyst exhibits the lowest Tafel slope and advantageous overpotentials at different current densities. The electron transfer number of HzOR was evaluated by RDE test at different rotation speeds to determine the electrooxidation degree of N_2_H_4_ (Fig. 5c). 6W–O–CoP/NF shows the electron transfer number of 3.35 close to the ideal value (4e^−^), suggesting a complete electrooxidation of N_2_H_4_ on [W–O]-doped CoP catalyst. Furthermore, the electroless “spontaneous decomposition” of N_2_H_4_ was also evaluated to investigate the electrochemical utilization ratio of N_2_H_4_ on obtained 6W–O–CoP/NF catalyst during HzOR (Figs. S23 and S24). The total electrooxidation efficiency of added N_2_H_4_ is calculated to be ~ 85.91%, suggesting that the strong interaction and dehydrogenation kinetics between the absorbed N_2_H_4_ and 6W–O–CoP/NF catalyst facilitate the electro-utilization of N_2_H_4_ during HzOR.

In order to investigate the intrinsic catalytic activity of 6W–O–CoP/NF toward HzOR, varied concentrations of N_2_H_4_ were measured. From Fig. S25, the current density sharply elevates with the increased N_2_H_4_ concentration, indicating that the mass transfer process is the rate-determining limit during HzOR. When the concentration reaches 0.1 M and above, the mass transfer limit could be largely overcome due to the porous and hierarchical nanoarrays of 6W–O–CoP/NF. Figure S26 displays the LSV curves of 6W–O–CoP/NF with different scan rates ranging from 1 to 100 mV s^−1^ in 0.1 M N_2_H_4_ electrolyte, where only slight changes could be detected, suggesting the efficient charge and mass transport process during HzOR. This is consistent with the EIS results in Fig. 5d and Table S5, where only a rather low charge transfer resistance (R_ct_) of 1.05 Ω could be fitted, much lower than those of other samples, demonstrating the fast electron transfer of 6W–O–CoP/NF during HzOR process. In addition, a comparison between the OER and HzOR performances of 6W–O–CoP/NF was also carried out as shown in Figs. 5e, f and S27, demonstrating the great kinetic advantages of HzOR electrocatalysis. It only requires lower potentials of − 50.65, − 34.74 and 19.84 mV in HzOR than those of OER (1.32, 1.62 and 1.75 V) to deliver the current densities of 10, 100 and 500 mA cm^−2^, respectively, indicating the potential application to replace OER for HzOR-assisted OWS. The long-term stability of 6W–O–CoP/NF toward HzOR was measured by chronoamperometry tests at overpotentials of − 22 mV (vs. RHE) (Fig. S28) and 28 mV (vs. RHE) (Fig. S29), and the current densities show the negligible decay. After 5,000 HzOR cycles, a slightly dropped overpotential (~ 7 mV@10 mA cm^−2^) can be observed (inset in Fig. S28), and the initial morphology and phase are mostly retained (Figs. S30–S32), indicating the good stability of 6W–O–CoP/NF catalyst in spite of the slight decrease of surface W(+ 6) amount.

To study the practical dehydrogenation process of W–O-CoP toward HzOR, the in situ Raman measurements was carried out. Compared with 1.0 M KOH electrolyte (Fig. 5g-left), the addition of 0.1 M N_2_H_4_ brings about the peak appearance at 1110, 1346 and 1602 cm^−1^, respectively, assigned to N–N bonds of absorbed ^*^N_2_H_4_ and N–H bonds of ^*^NH_2_, demonstrating the spontaneous adsorption of N_2_H_4_ molecules on the surface of WO–CoP, agreeing well with the DFT calculation result in Fig. 1e, f [74, 75]. When applying an ultra-small voltage (− 0.05 V), the peak density of N–H bonds in ^*^NH_2_ dramatically increases, indicating the accumulation of ^*^NH_2_ at this overpotential (including ^*^N_2_H_4_, ^*^N_2_H_3_, ^*^N_2_H_2_ intermediates). Impressively, with the increase in applied voltage from − 0.05 to 0 V, the ^*^NH_2_ peaks at 1346 and 1602 cm^−1^ gradually decrease, verifying that the accumulated ^*^NH_2_-based intermediates are dehydrogenated efficiently at such a low overpotential to produce ^*^N_2_H and ^*^N_2_ consequently releasing N_2_ to re-expose the catalytic active sites of WO–CoP, in accordance with the ultra-low Tafel slope (Fig. 5b) as well as the presence of PDS in WO-doped CoP (^*^N_2_H_2_ → ^*^N_2_H, Fig. 1f).

Owing to the excellent HER and HzOR catalytic performance, an OHzS unit with two-electrode system was assembled by applied 6W–O–CoP/NF as both anode and cathode electrodes catalysts (Fig. S33a). As shown in Fig. 5h, the OHzS unit possess significantly enhanced catalytic activity in comparison with OWS unit, only requiring the voltages of 8.73, 165.95 and 276.88 mV to achieve 10, 100 and 300 mA cm^−2^, respectively, which are 1.611, 1.634 and 1.713 V lower than the water splitting system (1.62, 1.80 and 1.99 V, respectively). For comparison, commercial 20 wt% Pt/C on Ni foam as both anode and cathode catalysts for the OHzS unit was also assembled (Fig. S33b), and the voltage of Pt/C||Pt/C is much higher than that of 6W–O–CoP/NF ||6W–O–CoP/NF, indicating the greatly reduced electricity consumption of the latter.

**Fig. 6:**
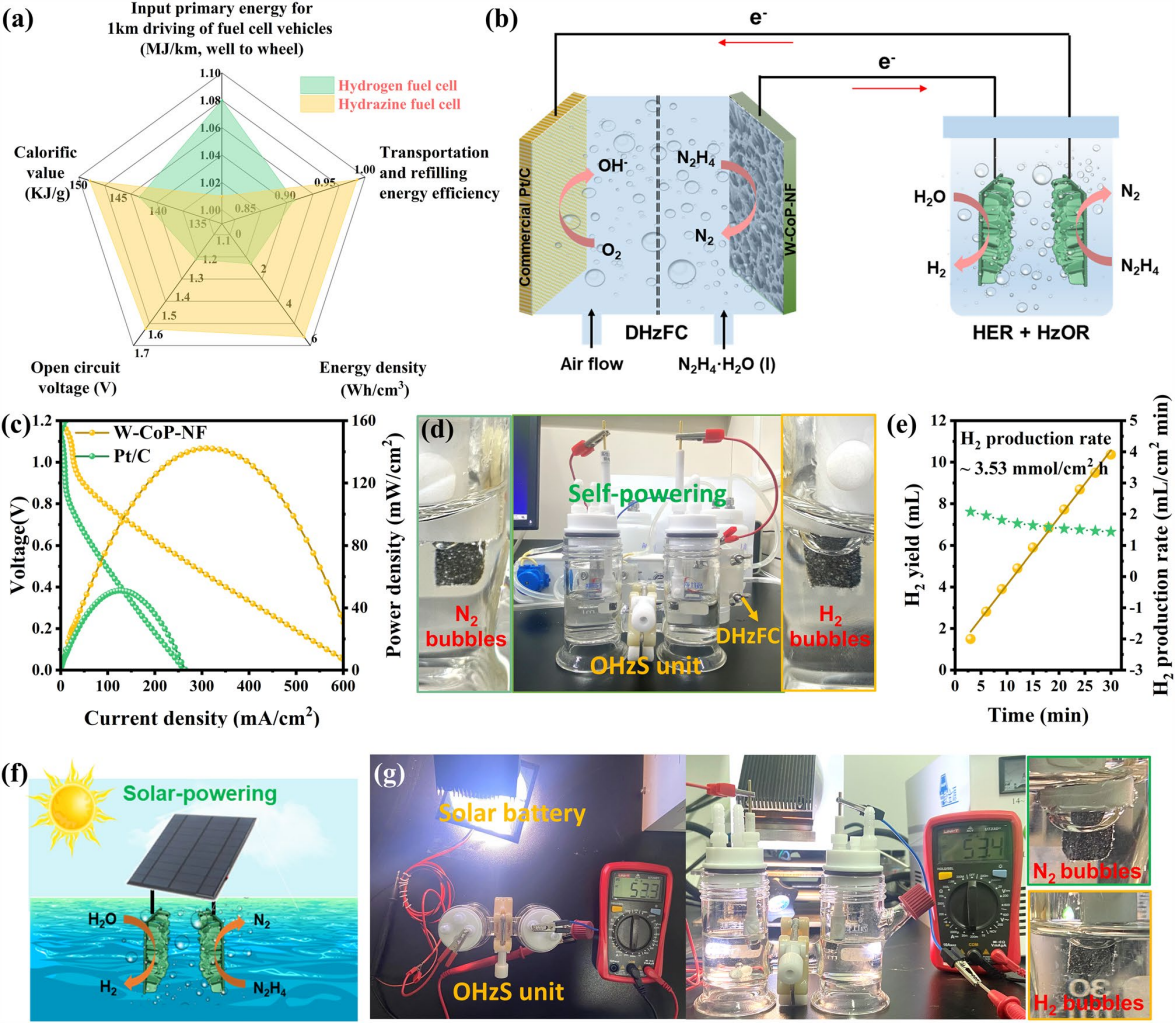
Two self-powered H_2_ production systems using 6W–O–CoP/NF electrodes. **a** Comparison of hydrogen and direct hydrazine fuel cells [74–77]. **b** Schematic illustration of a DHzFC-powered and HzOR-coupled H_2_ production system integrating a homemade DHzFC to drive the OHzS unit. **c** Discharge polarization curves and power density plots for the DHzFC in 1 M KOH + 0.1 M N_2_H_4_ electrolyte at room temperature. **d** Digital photograph of the integrated H_2_ production system powered by DHzFC. **e** The H_2_ generation yield and rate of the DHzFC-powered H_2_ production system. **f** Schematic illustration of the solar-powered OHzS in 1 M KOH + 0.1 M N_2_H_4_ electrolyte driven by a commercial solar cell and **g** the corresponding digital photograph of the evolution of gas bubbles on 6W–O–CoP/NF || 6W–O–CoP/NF

In order to further demonstrate the potential application, a self-powered system by integrating a DHzFC using 6W–O–CoP/NF as anode and commercial Pt/C as cathode, to drive the OHzS unit catalyzed by 6W–O–CoP/NF at both anode and cathode, has been assembled (Fig. 6a, b). In the homemade DHzFC, 6W–O–CoP/NF was used as the anode in 1 M KOH + 0.1 M N_2_H_4_ electrolyte, and the commercial 20% Pt/C coated on composite substrate was used as cathode in 1 M KOH electrolyte. For comparison, the DHzFC catalyzed by commercial 20% Pt/C in both anode and cathode was also assembled. It is worth noting that the DHzFC equipped with 6W–O–CoP/NF exhibits a high OCV of 1.1 V (Fig. S34) and a maximal power density (*P*_max_) of 142 mW cm^−2^ (Fig. 6c) at room temperature, which is 2.8 fold that of commercial Pt/C based cell (*P*_max_ = 50 mW cm^−2^), and also superior to those of the state-of-the-art values (Table S6). Moreover, the homemade DHzFC can run stably at the current densities of 1, 2, 3, 4, 5 mA cm^−2^ for 25 h without significant decay (Fig. S35). Owing to the high output, two in series assembled DHzFCs can power a LED display (1.5 V) or a light-emitting diode (2–2.2 V) (Fig. S36). As a proof-of-concept, the self-powered H_2_ production system is established by integrating the DHzFC to drive OHzS unit (Fig. 6d), where the vigorous generation of gas bubbles on the 6W–O–CoP/NF electrode surface can be visualized (Video S1). An excellent H_2_ production rate of 3.53 mmol cm^−2^ h could be achieved at room temperature powered by the DHzFC (Fig. 6e). Finally, the capability of the 6W–O–CoP/NF catalyst for catalyzing solar energy-driven OHzS was also determined by applying a commercial polycrystalline silicon solar cell (5.5 V, 1 W) under the irradiation of Xenon lamp (Fig. 6f). The solar cell could achieve a high current output of ~ 53.4 mA, and the continuous bubbles could be observed at both anode and cathode, suggesting the feasibility of the solar-energy-driven hybrid water splitting on 6W–O–CoP/NF electrode (Fig. 6g).

## Conclusions

In summary, the [W–O] species are introduced into CoP nanoflakes (denoted as 6W–O–CoP/NF) via the hydrolysis etching and doping strategy, which not only works as the effective absorption sites for H_2_O and N_2_H_4_ molecules to accelerate the dissociation (Volmer step) and dehydrogenation, respectively, but also enables the porous nanostructure formation as well as the electronic modification of CoP nanoflakes to promote the Heyrovsky step during HER. Specifically, the obtained 6W–O–CoP/NF could achieve the current density of 1000 mA cm^−2^ at a low overpotential of 185.6 mV for alkaline HER, and only requires 78.99 mV to reach 1000 mA cm^−2^ for HzOR with a low Tafel slope of 8.43 mV dec^−1^ and nearly 4-electon process, which are better than most reported non-noble metal or noble metal catalysts. The electrolyzer equipped with 6W–O–CoP/NF as both anode and cathode catalysts could offer the current density of 100 mA cm^−2^ at a low cell voltage of 0.165 V for H_2_ production, greatly reducing the electric consumption. Such a HER/HzOR electrolyzer could be driven by two in series assembled DHzFC to realize the self-powered H_2_ production at a rate up to 3.53 mmol cm^−2^ h^−1^ without any external electricity.

### Supplementary Information

Below is the link to the electronic supplementary material.

Supplementary file1 (MP4 6384 KB)

Supplementary file2 (PDF 3923 KB)
