# Supplementary material for: Adsorption Site Regulations of [W–O]-Doped CoP Boosting the Hydrazine Oxidation-Coupled Hydrogen Evolution at Elevated Current Density
Source: Nanomicro Lett. 2023 Sep 14;15:212. doi: 10.1007/s40820-023-01185-4 (PMC10501108; doi:10.1007/s40820-023-01185-4)
Supplement: Supplementary file 2 — Supplementary file2 (PDF 3923 KB) [file 40820_2023_1185_MOESM2_ESM.docx]

Supporting Information (SI)

**Adsorption site regulations of [W-O]-doped CoP boosting the hydrazine oxidation-coupled hydrogen evolution at elevated current density**

Ge Meng^1, 2^, Ziwei Chang^1^, Libo Zhu^1, 2^, Chang Chen^1, 2^, Yafeng Chen^1^, Han Tian^1^, Wenshu Luo^1, 2^, Wenping Sun^3^, Xiangzhi Cui^1, 2, 4,^ *, and Jianlin Shi^1, 2,^ *

Ge Meng and Ziwei Chang contributed equally.

*Email: cuixz@mail.sic.ac.cn, jlshi@mail.sic.ac.cn.

1. **Supplementary section**

**
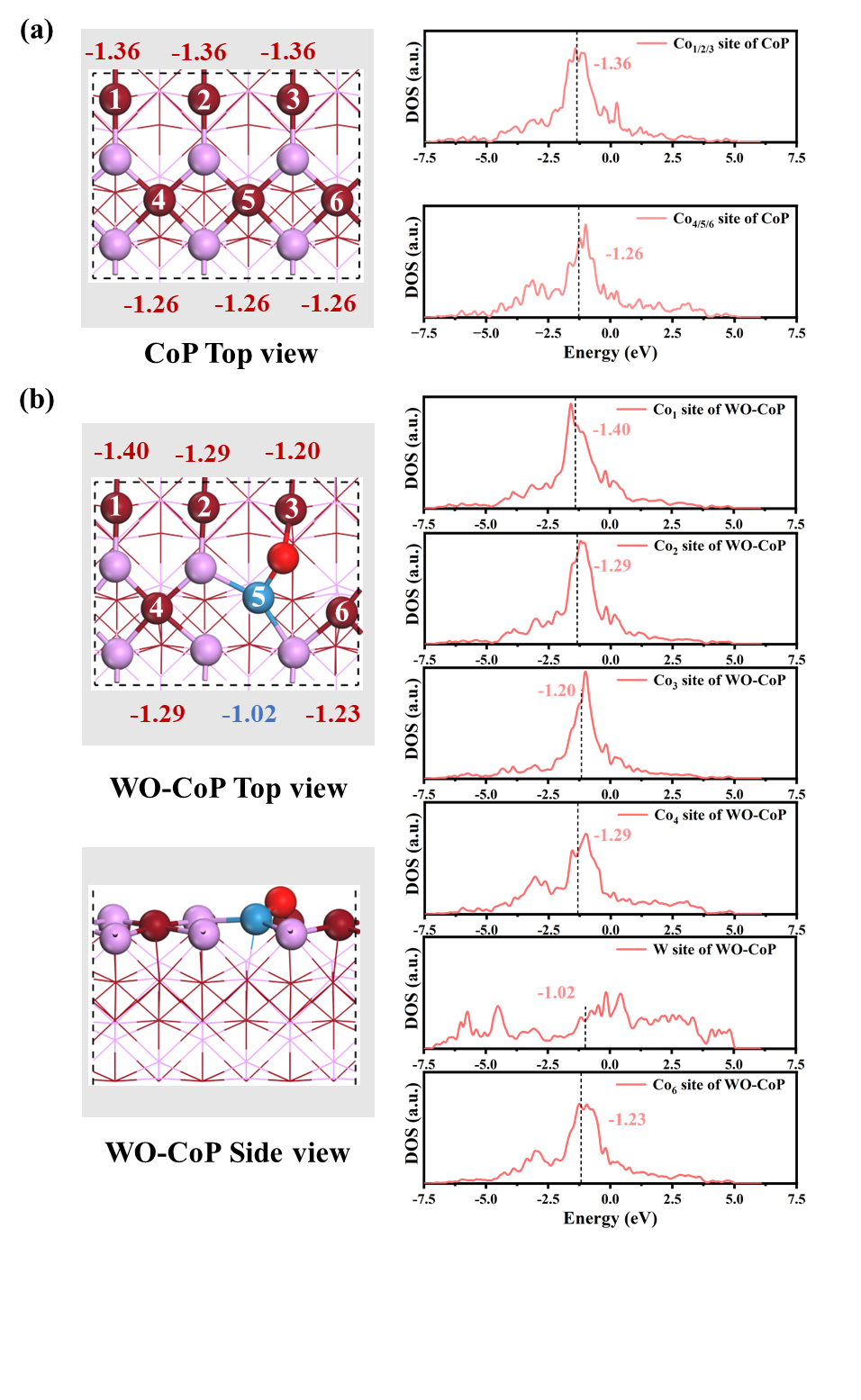
**

**Figure S1.** The calculated d-band center values of different sites in (a) pure CoP and (b) WO-CoP. (Co-Brownish red, P-pink atom, W-blue atom, O-red atom)

**
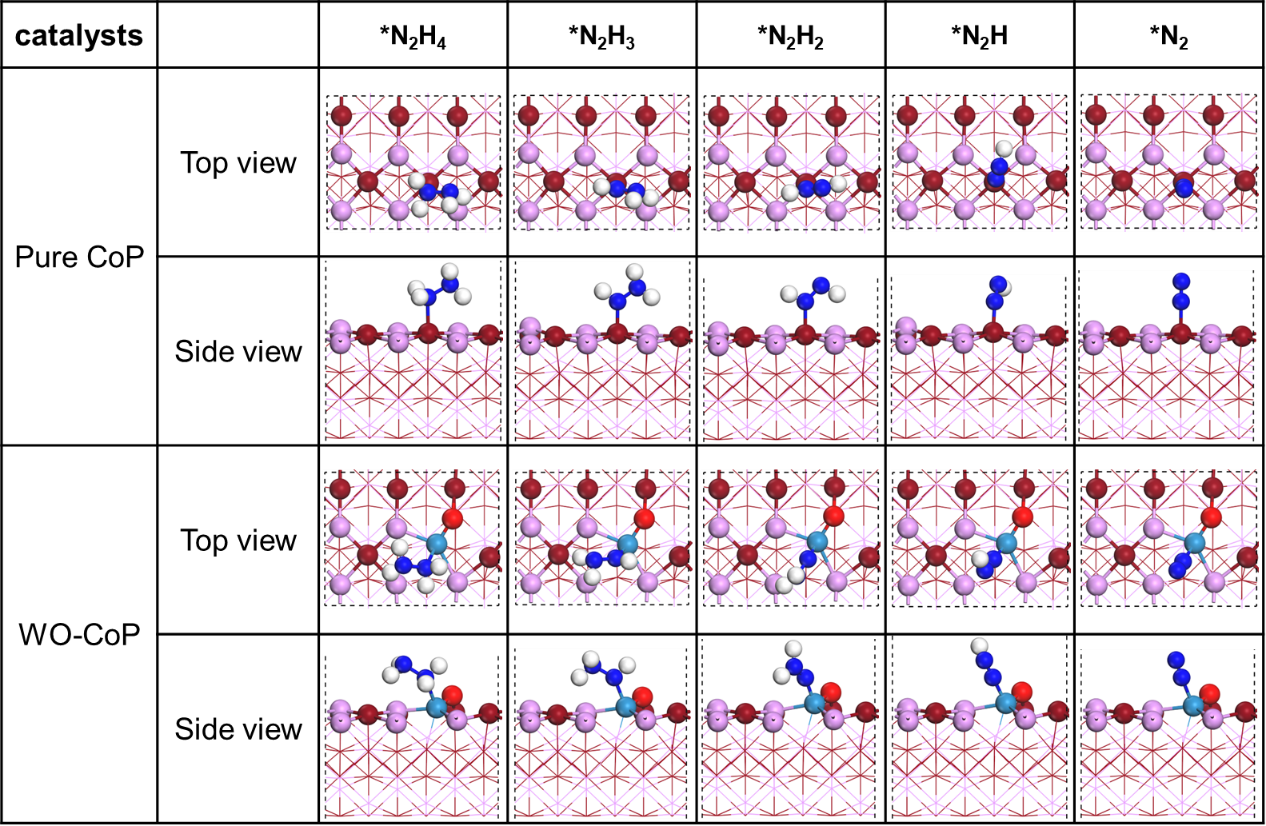
**

**Figure S2.** The optimized atomic structures of intermediates during N_2_H_4_ oxidation on the pure CoP (011) and W-O doped CoP (011) surface. (Co-Brownish red, P-pink atom, W-blue atom, O-red atom, H-white atom, N-dark blue atom)

**
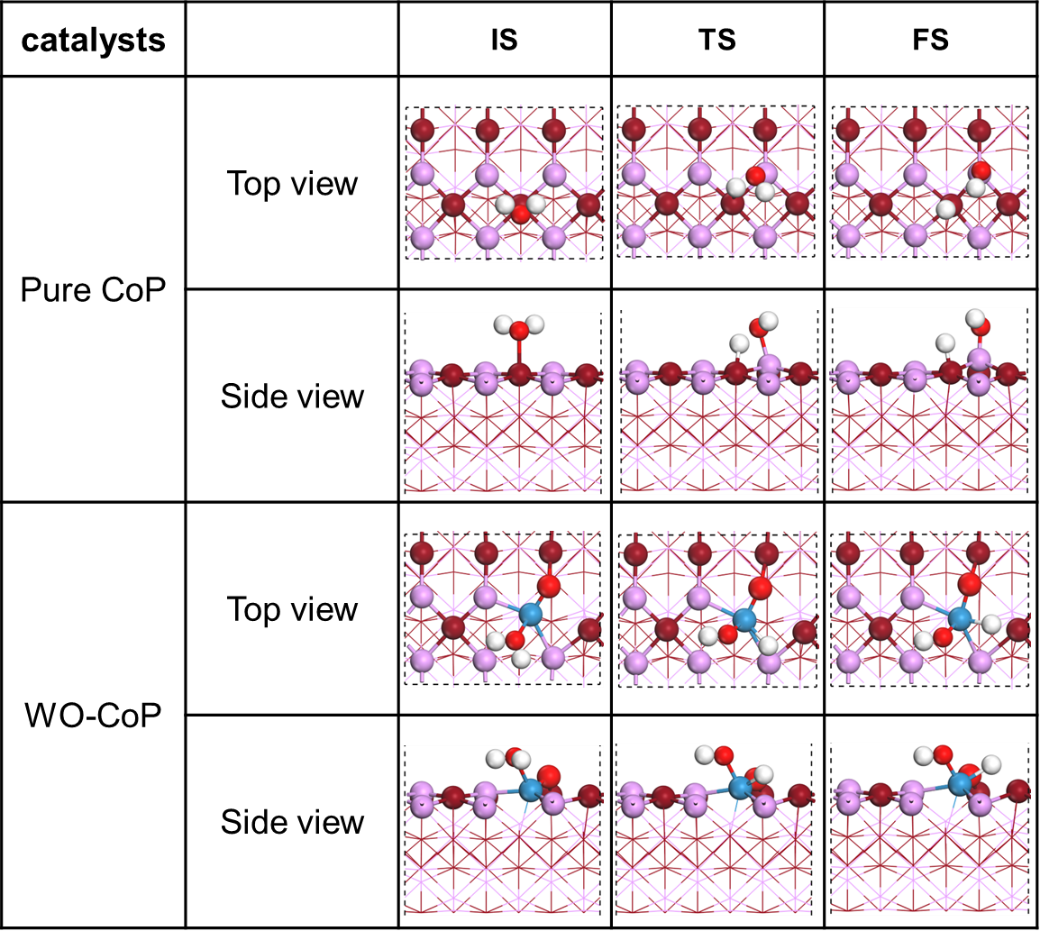
**

**Figure S3.** The optimized atomic structures of intermediates during water dissociation on the pure CoP (011) and W-O doped CoP (011) surface. (Co-Brownish red, P-pink atom, W-blue atom, O-red atom, H-white atom)

**
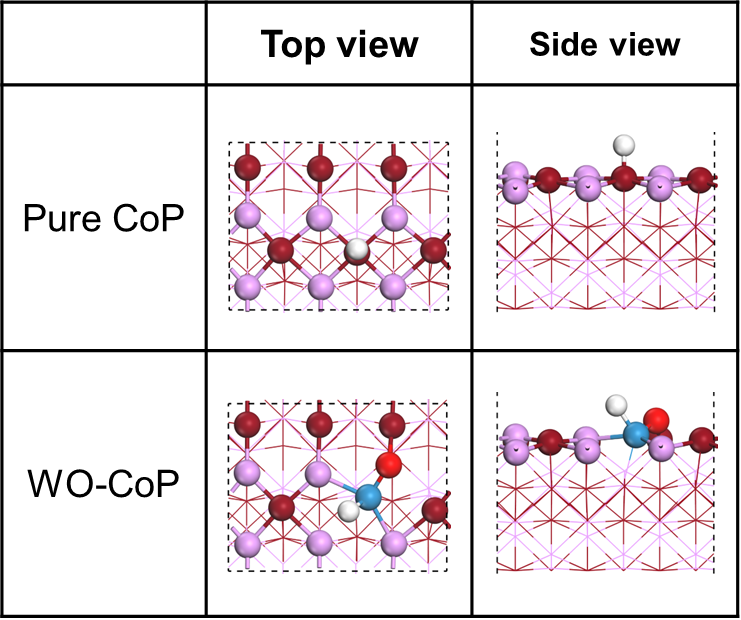
**

**Figure S4.** The optimized atomic structures of H_ads_ during hydrogen adsorption on the pure CoP (011) and W-O doped CoP (011) surface. (Co-Brownish red, P-pink atom, W-blue atom, O-red atom, H-white atom)

**
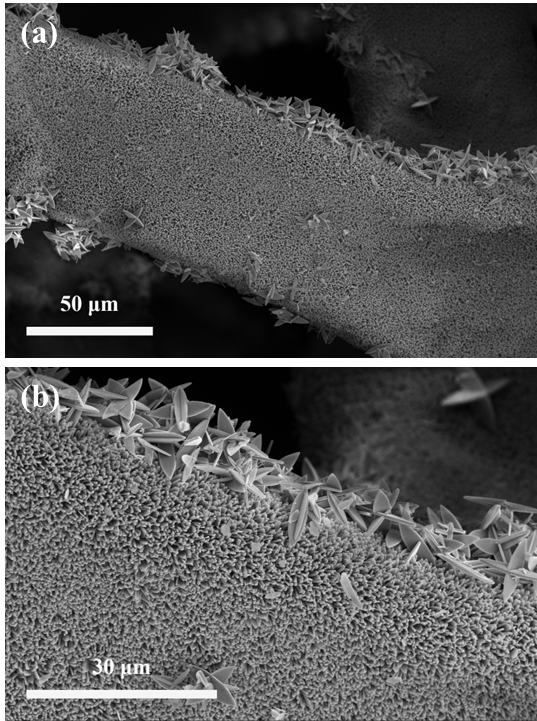
**

**Figure S5.** (a, b) SEM images of ZIF-L/NF precursor at different magnifications.

**
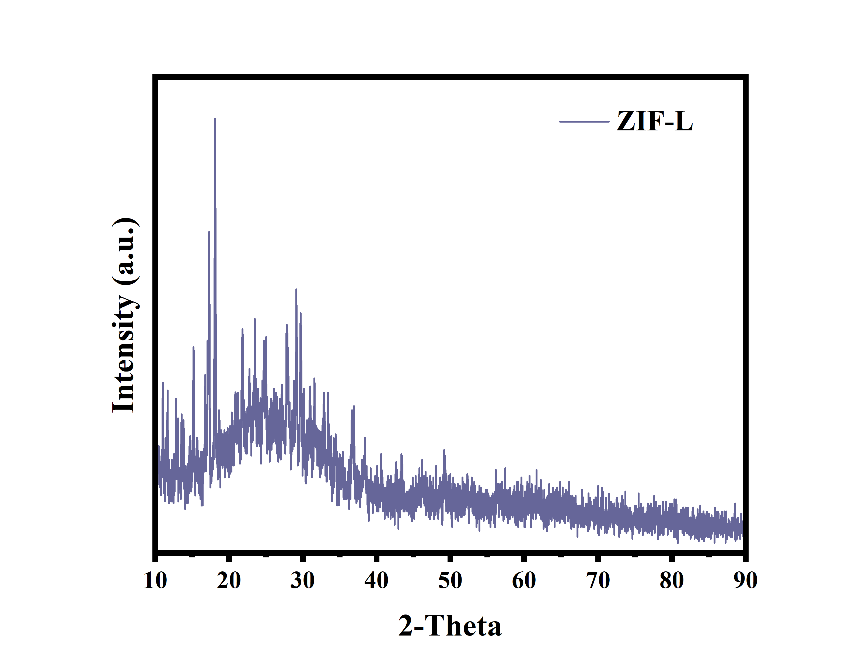
**

**Figure S6.** XRD pattern of ZIF-L.

**
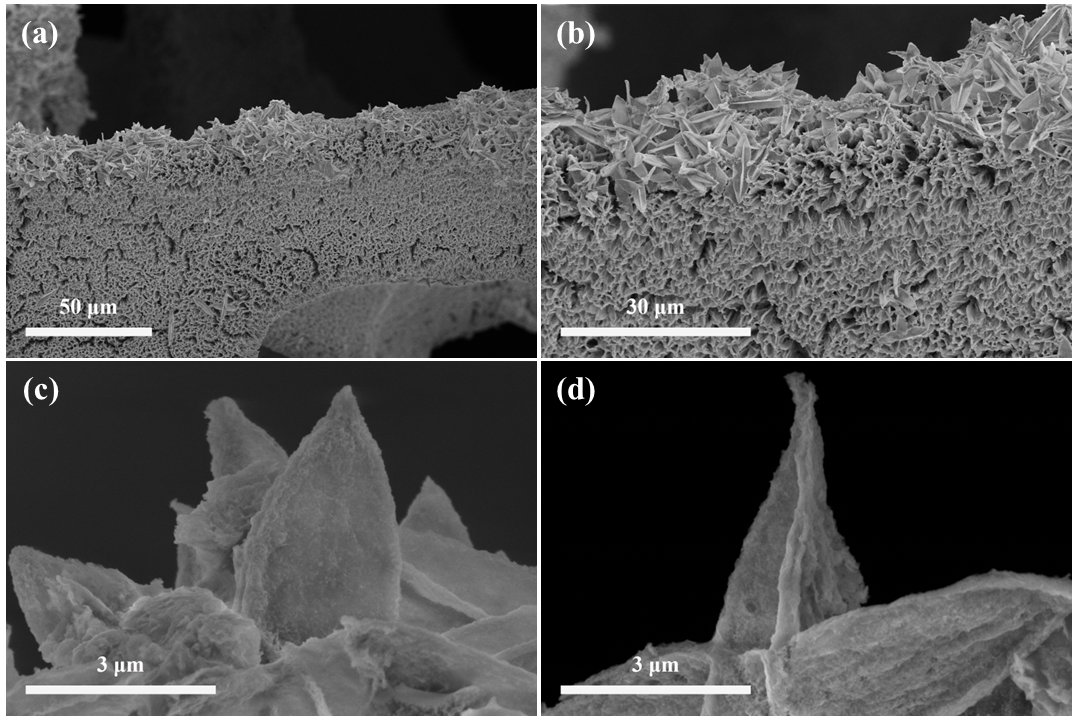
****Figure S7.** (a-d) SEM images of 6W-O-Co/NF at different magnifications.

**
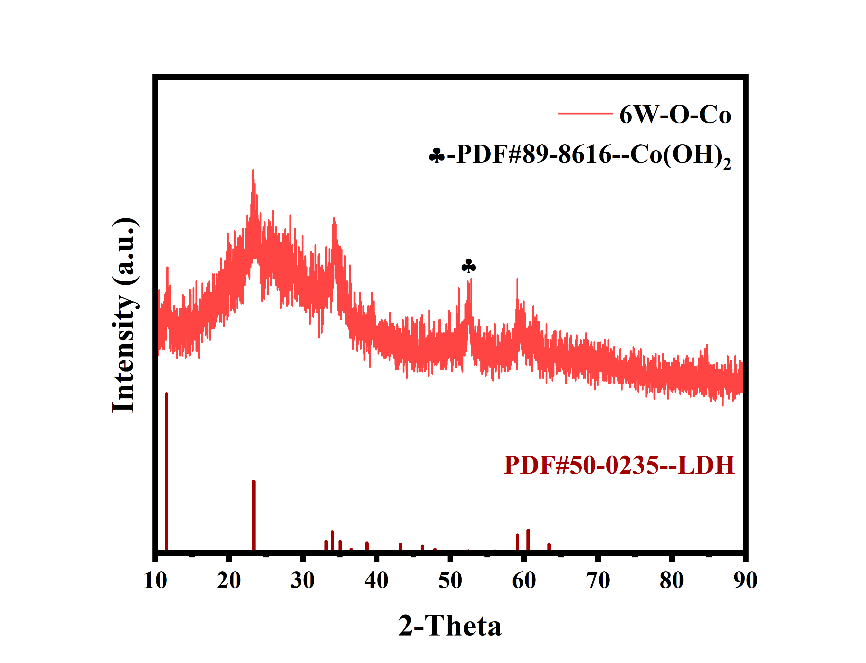
**

**Figure S8.** XRD pattern of 6W-O-Co.

**
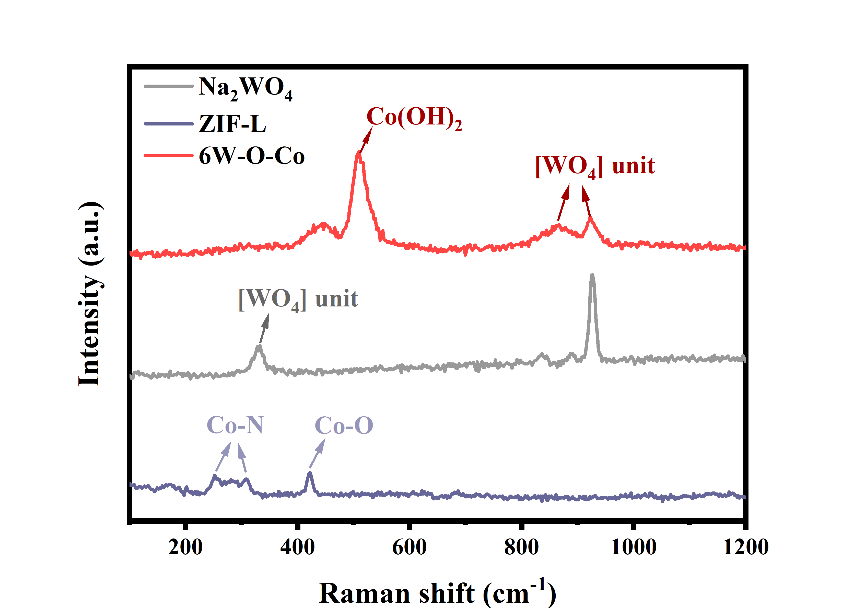
**

**Figure S9.** Raman spectra of 6W-O-Co.

**
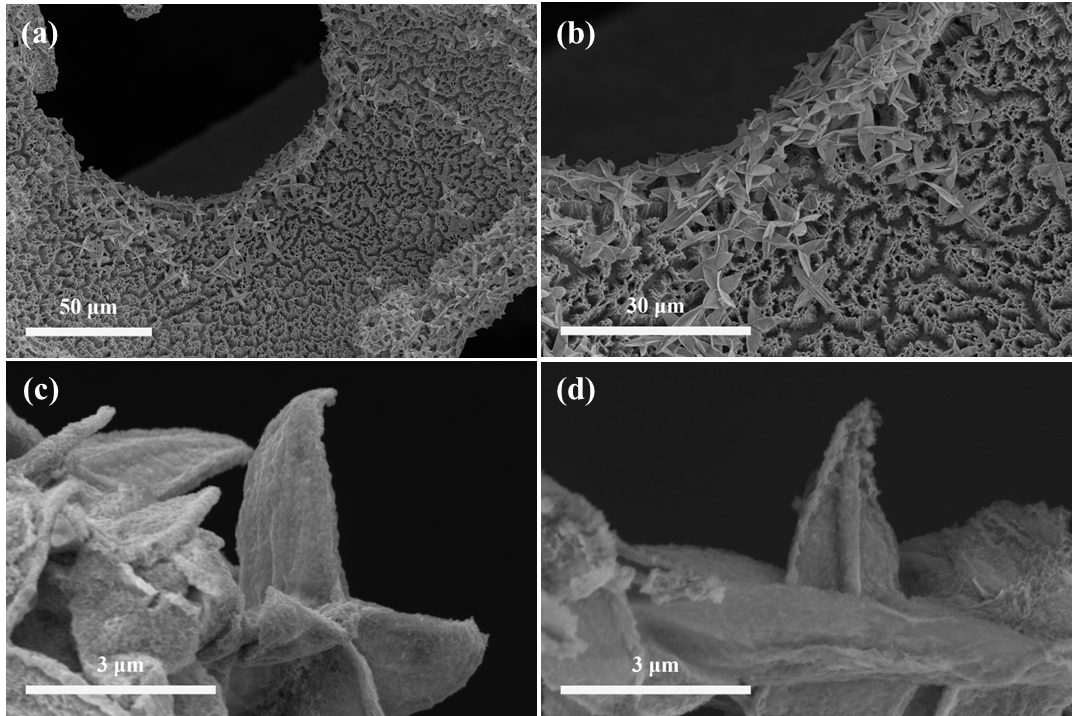
**

**Figure S10.** (a-d) SEM images of 6W-O-CoP/NF at different magnifications.

**
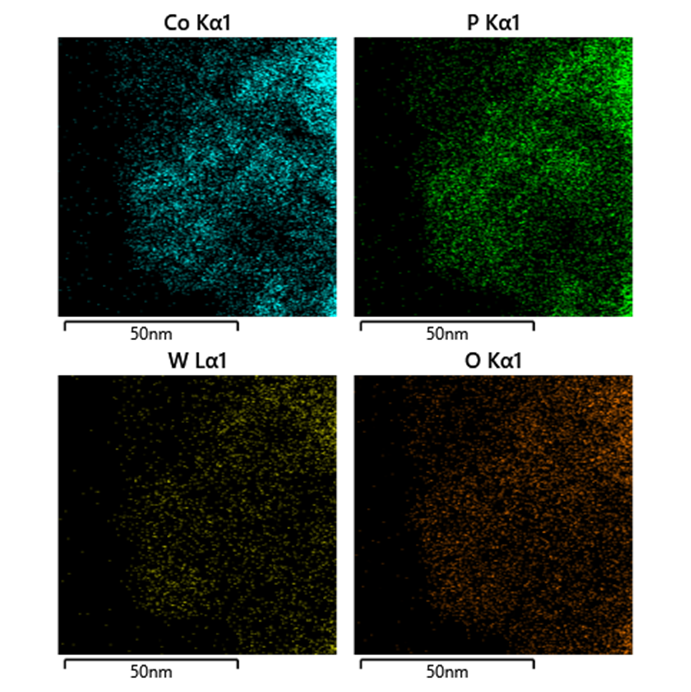
**

**Figure S11.** EDS mapping images of 6W-O-CoP/NF.


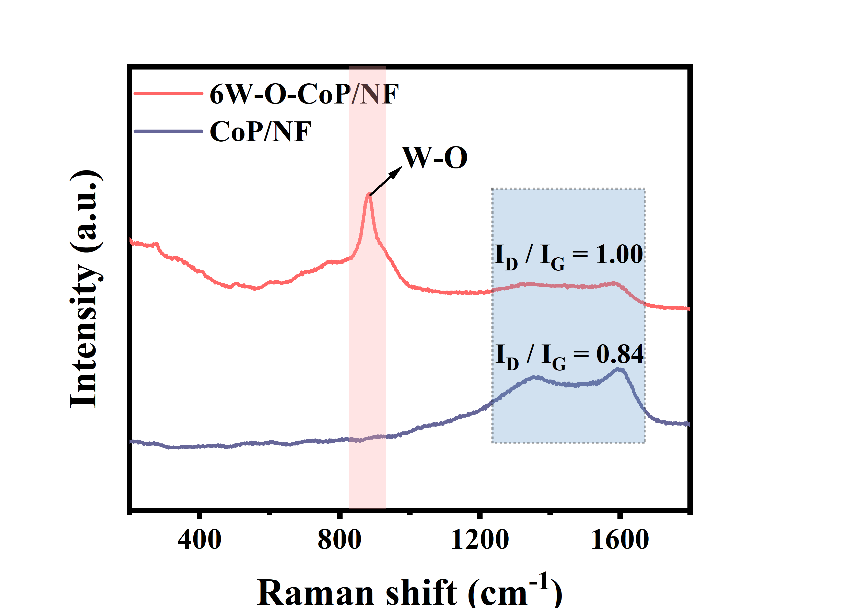


**Figure S12.** Raman spectra of 6W-O-CoP/NF and CoP/NF without etching and doping treatment.

**
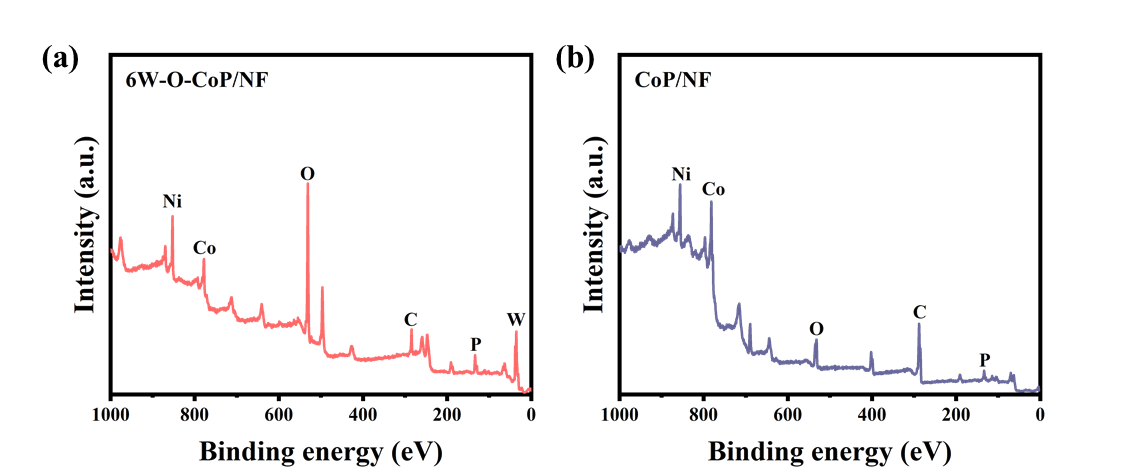
**

**Figure** **S13.** XPS full scan spectra of (a) 6W-O-CoP/NF and (b) CoP/NF.

**
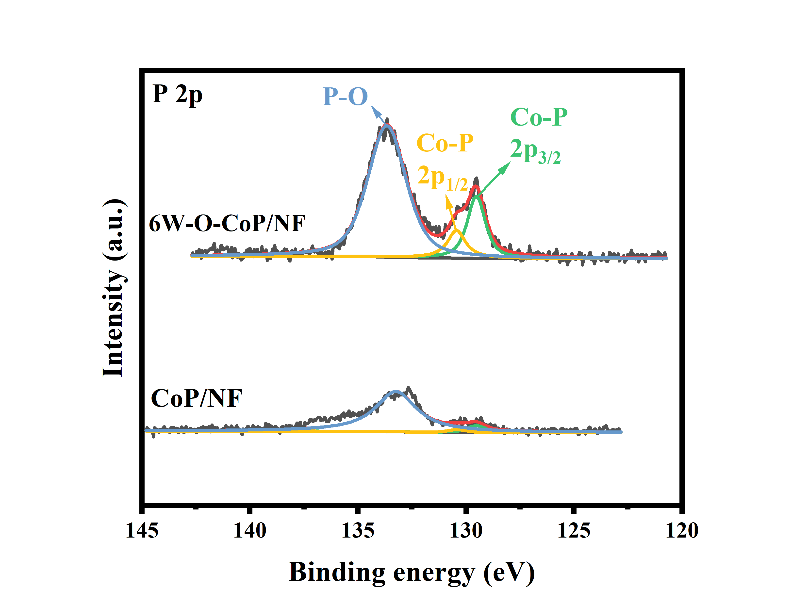
**

**Figure S14.** High-resolution XPS spectra of P 2p in the prepared samples.

**
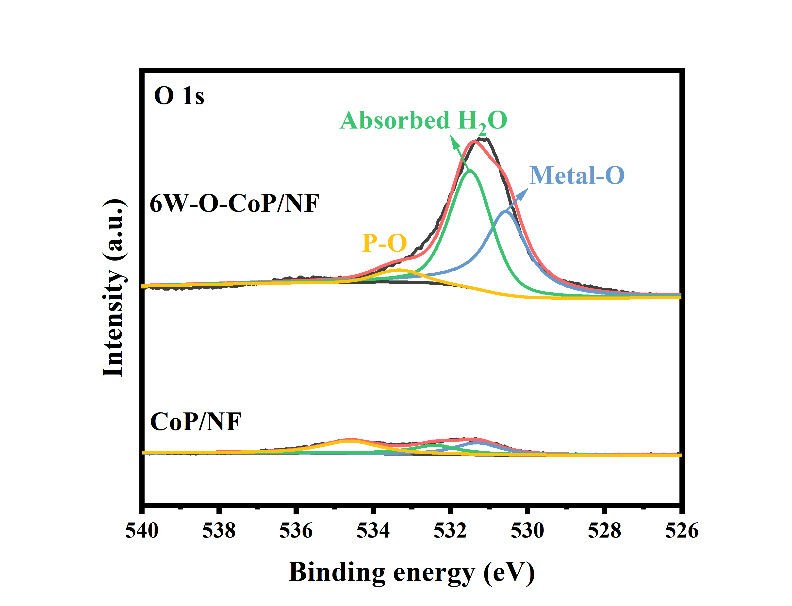
**

**Figure S15.** High-resolution XPS spectra of O 1s in the prepared samples.

**
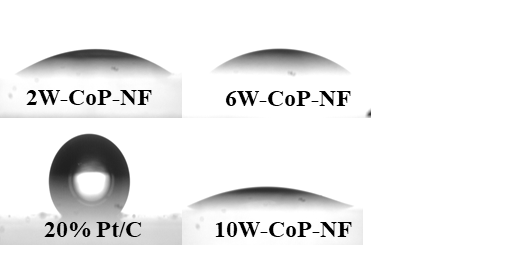
**

**Figure S16.** Water bubble contact angle measurement of the obtained catalysts.

**
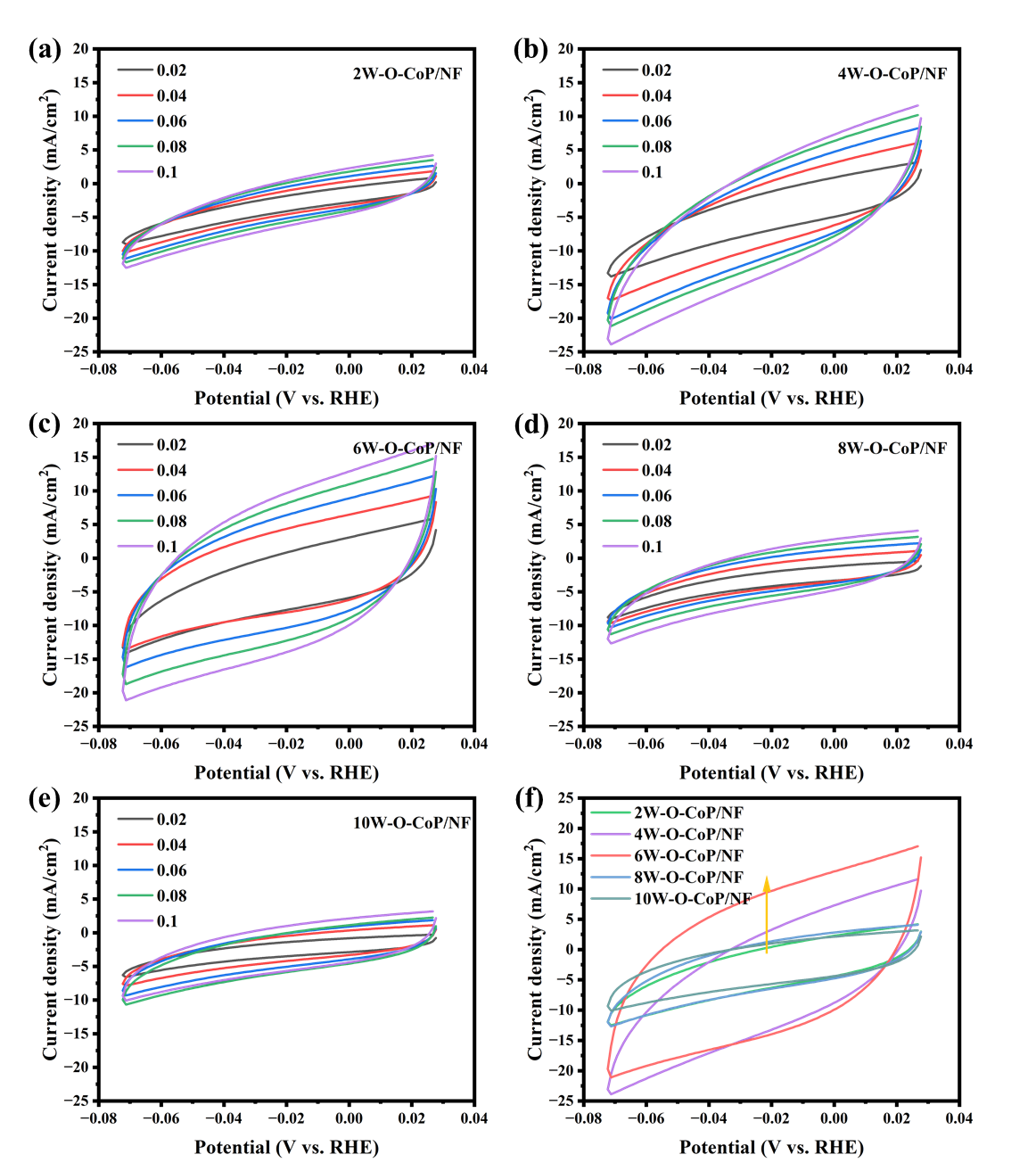
**

**Figure S17.** Cyclic voltammograms (CVs) of (a) 2W-O-CoP/NF, (b) 4W-O-CoP/NF, (c) 6W-O-CoP/NF, (d) 8W-O-CoP/NF and (e) 10W-O-CoP/NF at varied scan rate in 1.0 M KOH. (f) The corresponding CVs of xW-O-CoP/NF based catalysts at the scan rate of 100 mV/s.


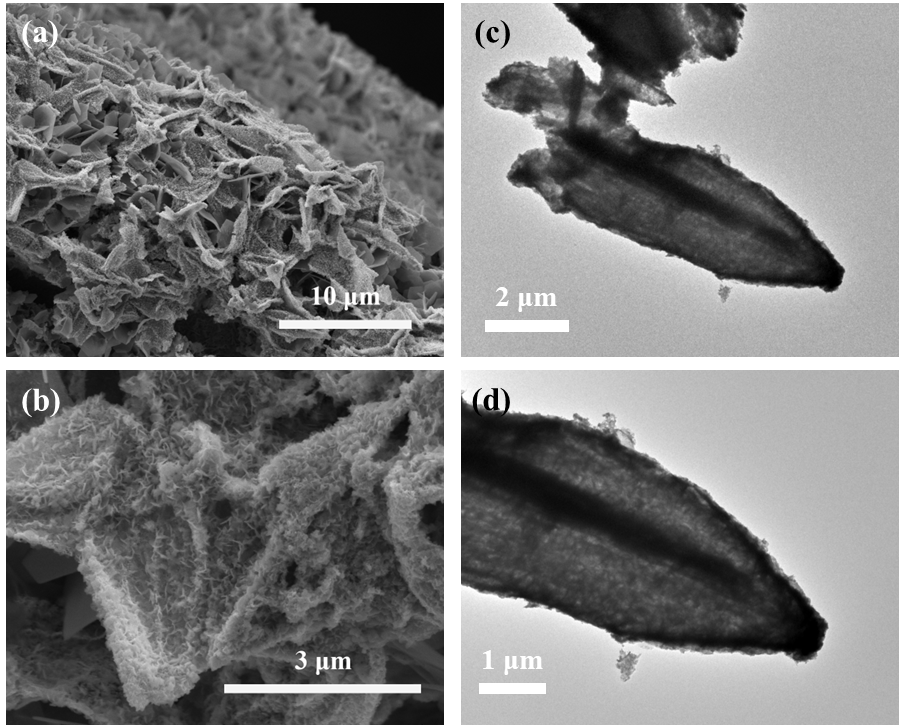


**Figure S18.** (a, b) SEM and (c, d) TEM images of 6W-O-CoP/NF catalyst after 10000 cycles of HER test at different magnifications.


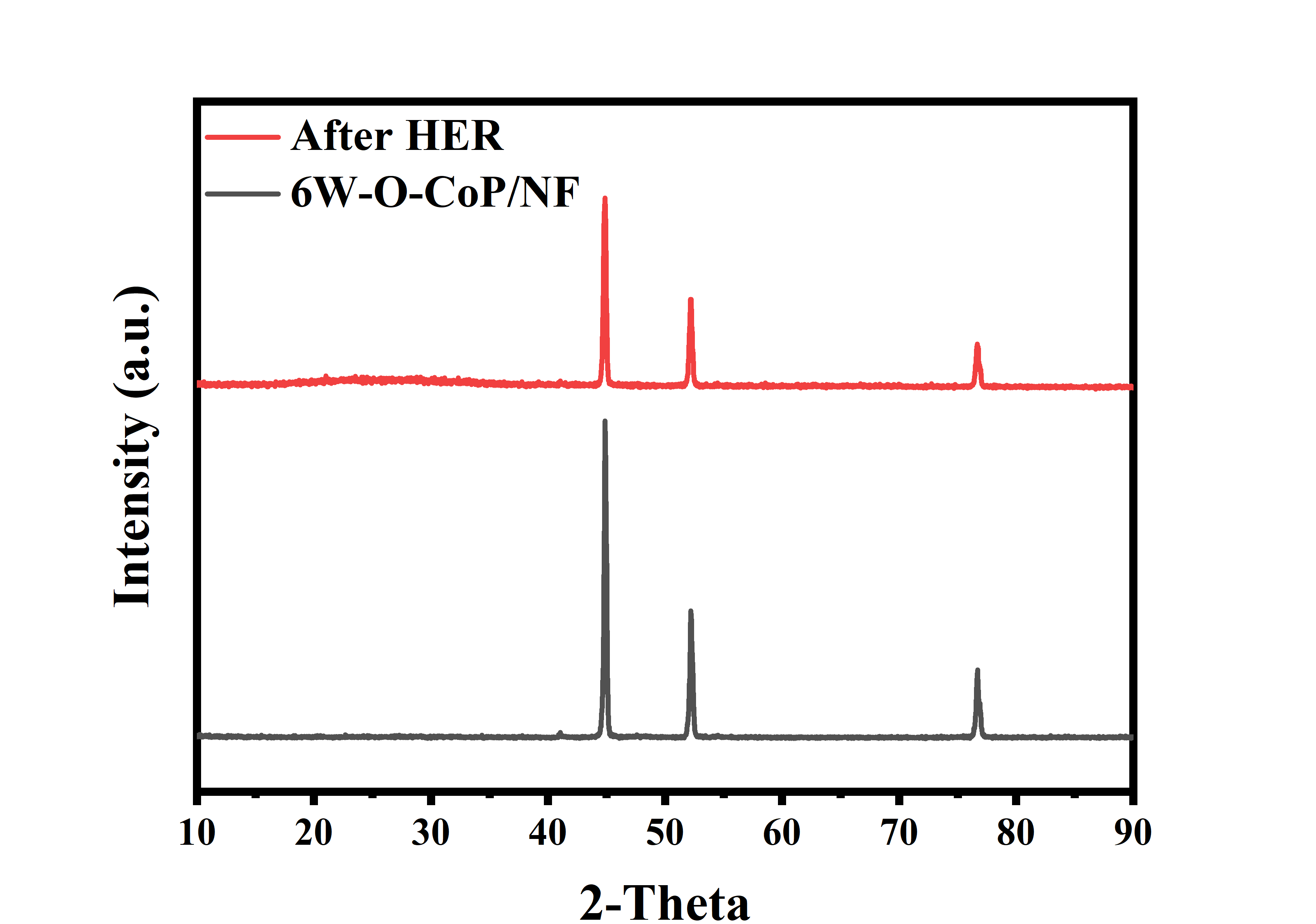


**Figure S19**. XRD patterns of 6W-O-CoP/NF catalyst before and after 10000 cycles of HER test.


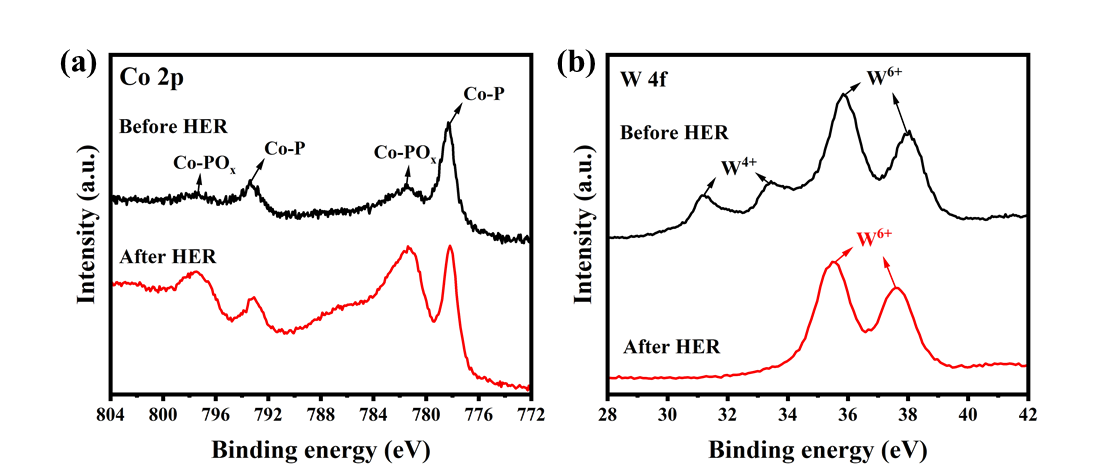


**Figure S20.** High resolution XPS spectra of (a) Co 2p and (b) W 4f for 6W-O-CoP/NF catalyst after HER 10000 cycles.


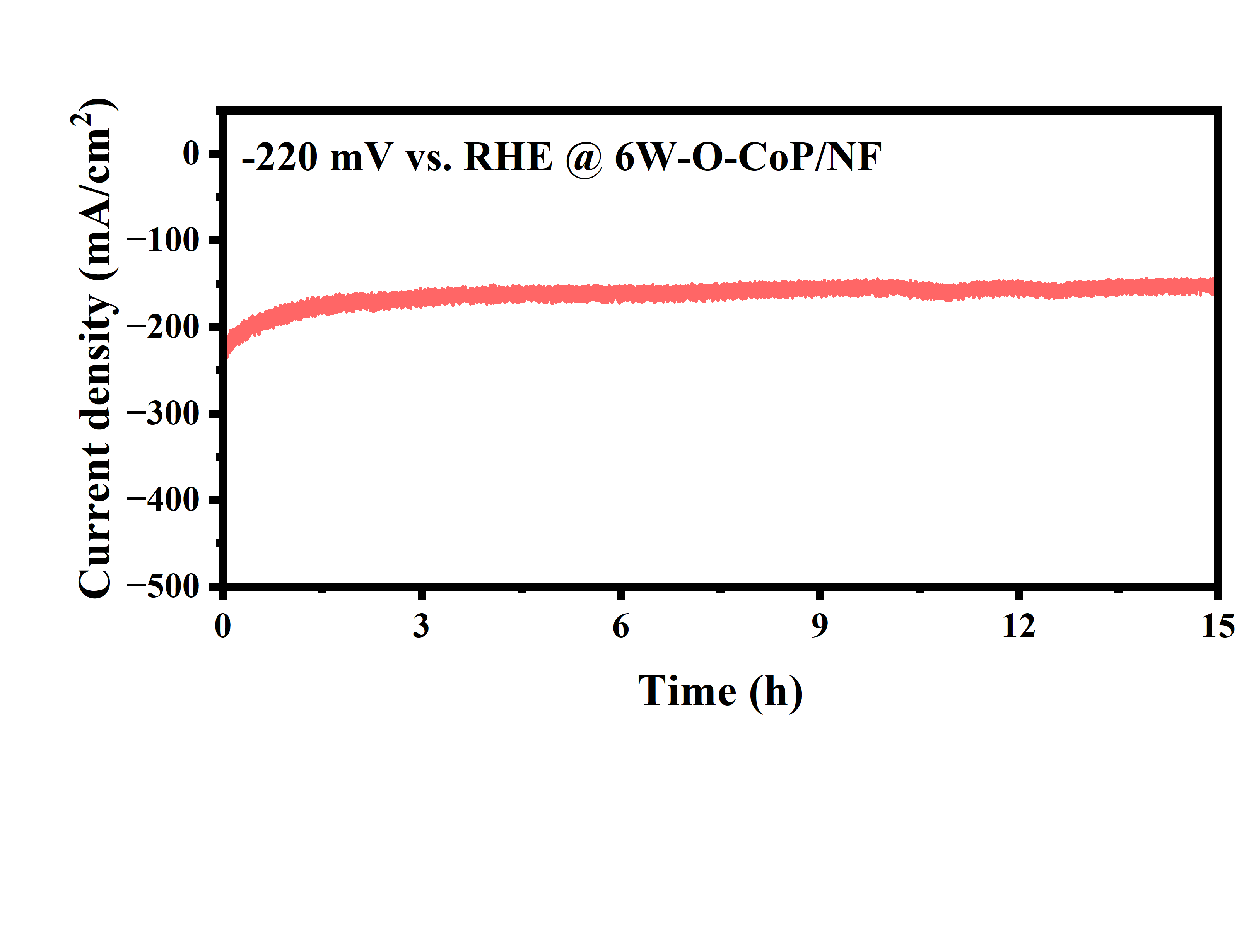


**Figure S21.** Stability measurement of 6W-O-CoP/NF at overpotential of -220 mV (vs. RHE) for 15 h.


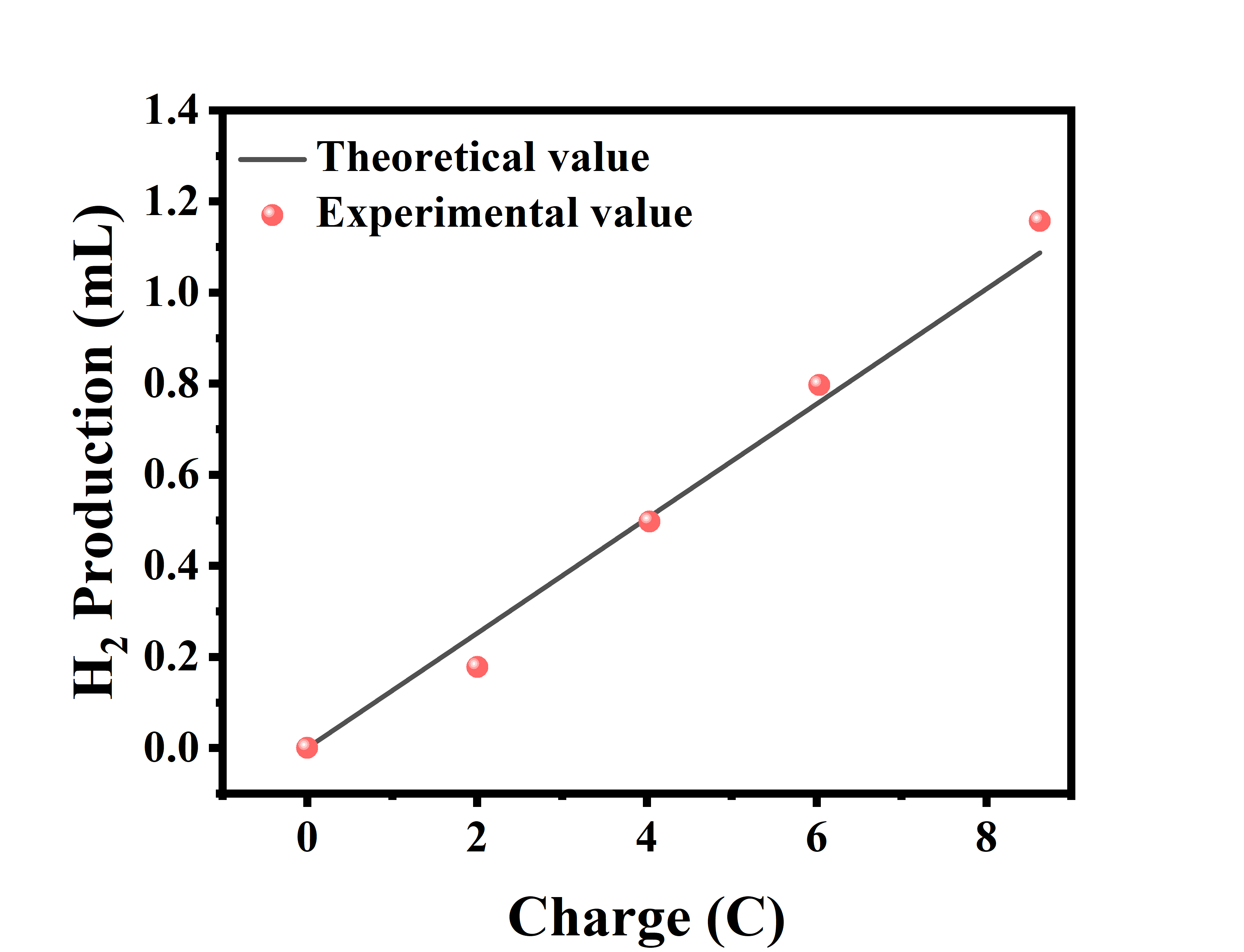


**Figure S22**. Comparison between GC-measured and theoretically calculated H_2_ quantities of 6W-O-CoP/NF at overpotential of -220 mV (vs. RHE).

**
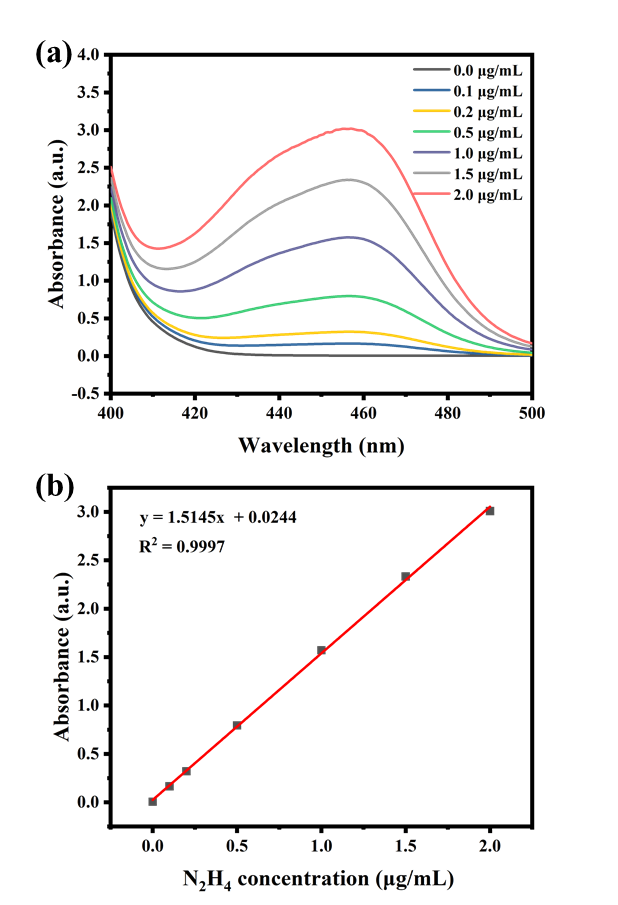
**

**Figure S23.** (a) UV-vis absorption spectra of different concentrations of hydrazine stained with color reagent. (b) Calibration curve for calculating the hydrazine concentration. The absorbance at 458 nm was measured by a UV-Vis spectrophotometer, and the fitting curve shows good liner relation of absorbance with N_2_H_4_ concentration (y = 1.5145x + 0.0244, R^2^ = 0.9997).

**
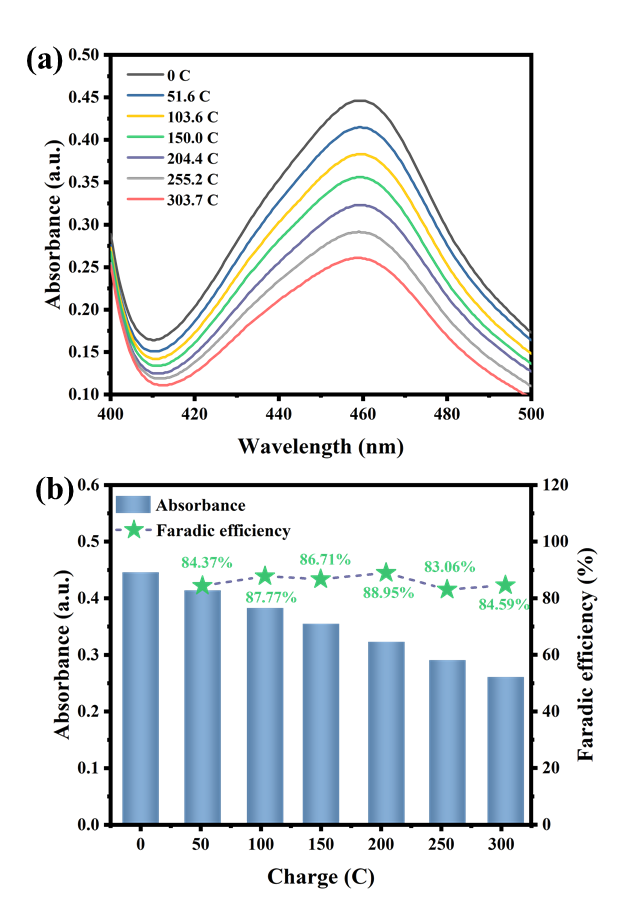
**

**Figure S24.** (a) UV-vis absorption spectra of hydrazine concentrations with different charge consumption during HzOR process. (b) The corresponding calculated utilization efficiency of N_2_H_4_.

HzOR measurement was conducted at constant potential. We would pause the test when reaching certain charge (0 C, 51.6 C, 103.6 C, 150.0 C, 204.4 C, 255.2 C and 303.7 C) and then record the time and extract the electrolyte from the reaction cell to measure the concentration of N_2_H_4_. Comparing the real and ideal concentration of N_2_H_4_ to determine the electro-oxidation efficiency of N_2_H_4_.

**
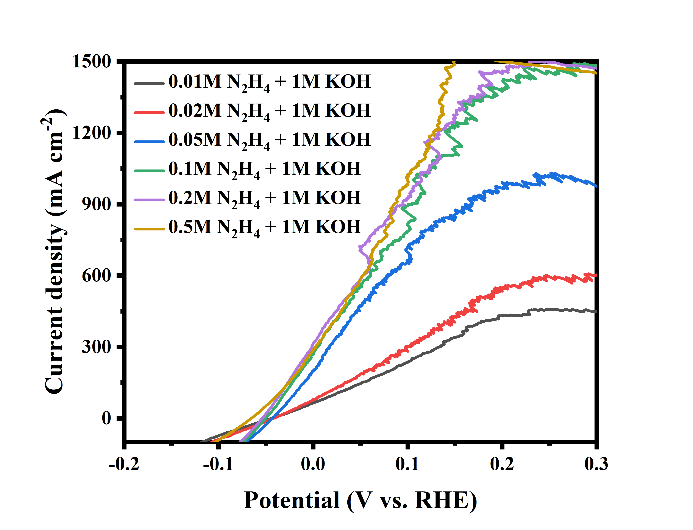
**

**Figure S25**. LSV curves of 6W-O-CoP/NF under different concentrations of hydrazine.

**
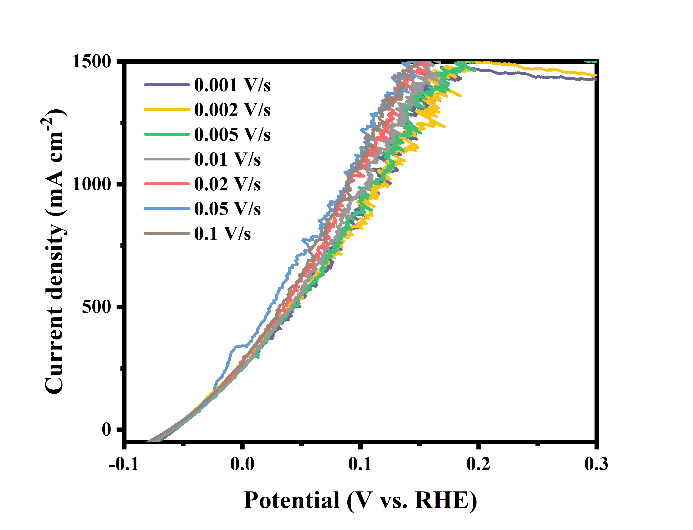
**

**Figure S26**. LSV curves of 6W-O-CoP/NF at different scan rate in 0.1M N_2_H_4_ + 1M KOH electrolyte.


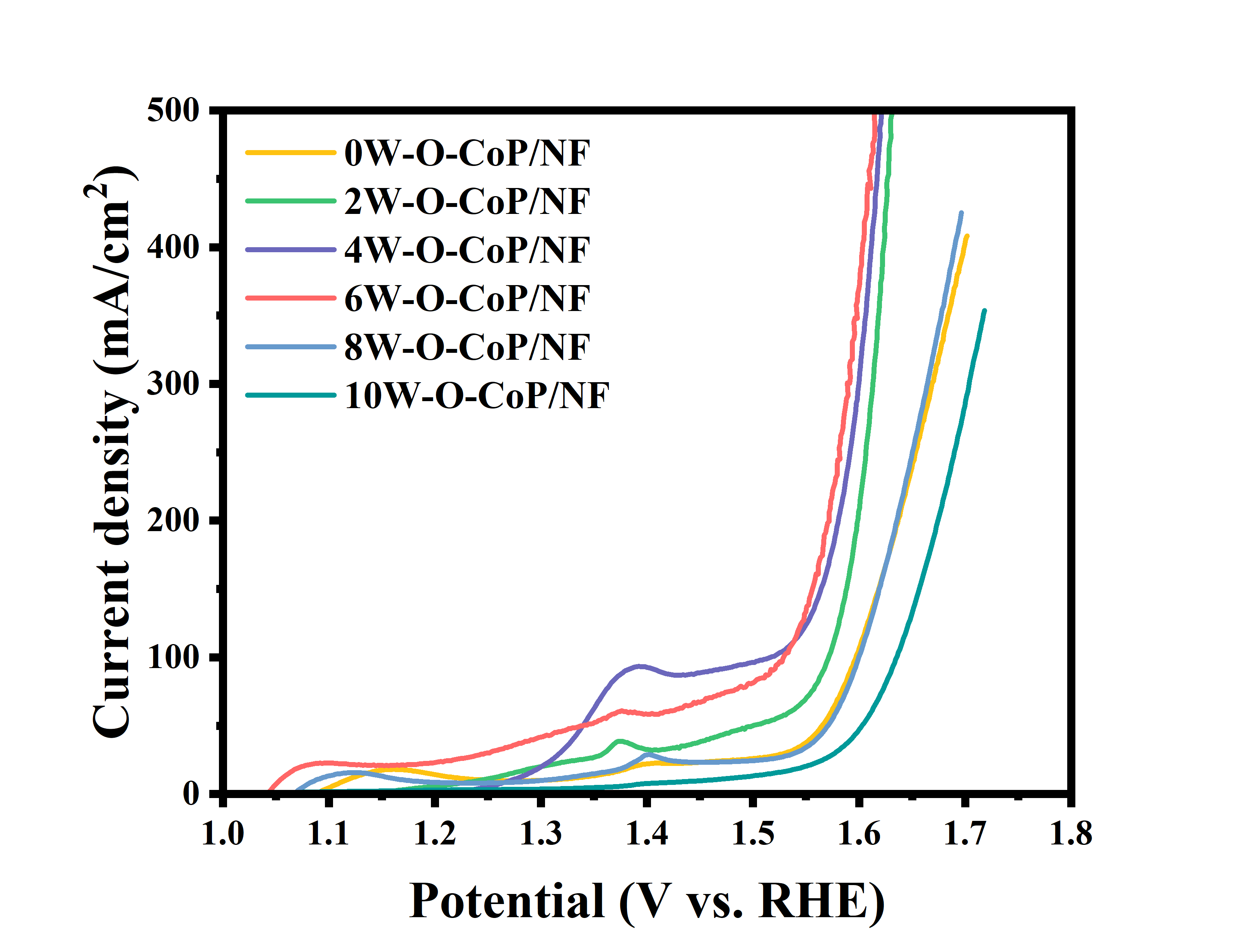


**Figure S27**. LSV curves of obtained xW-O-CoP/NF catalysts in 1.0M KOH for OER.


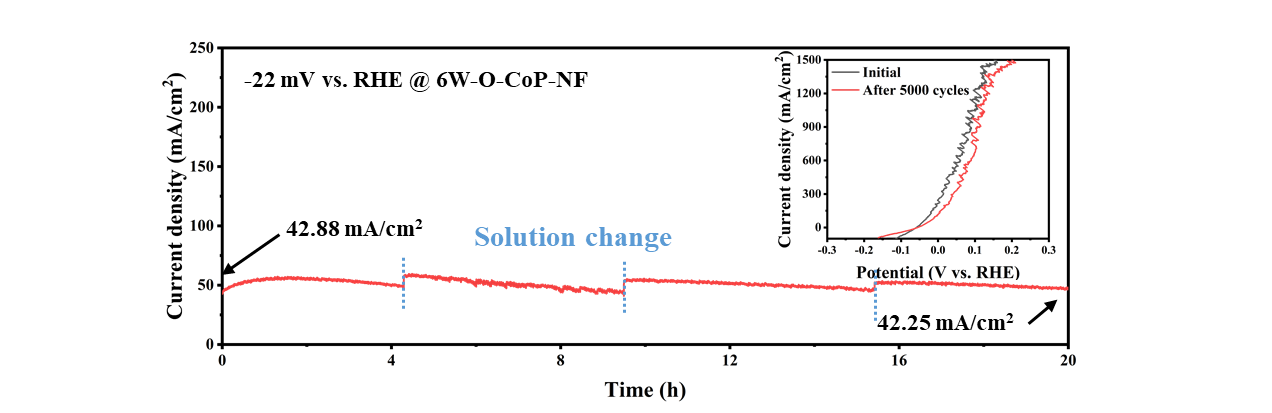


**Figure S28.** Stability measurement for 6W-O-CoP/NF at overpotential of -22 mV (vs. RHE) for 20 h (inset: the LSV curves before and after 5000 HzOR cycles).


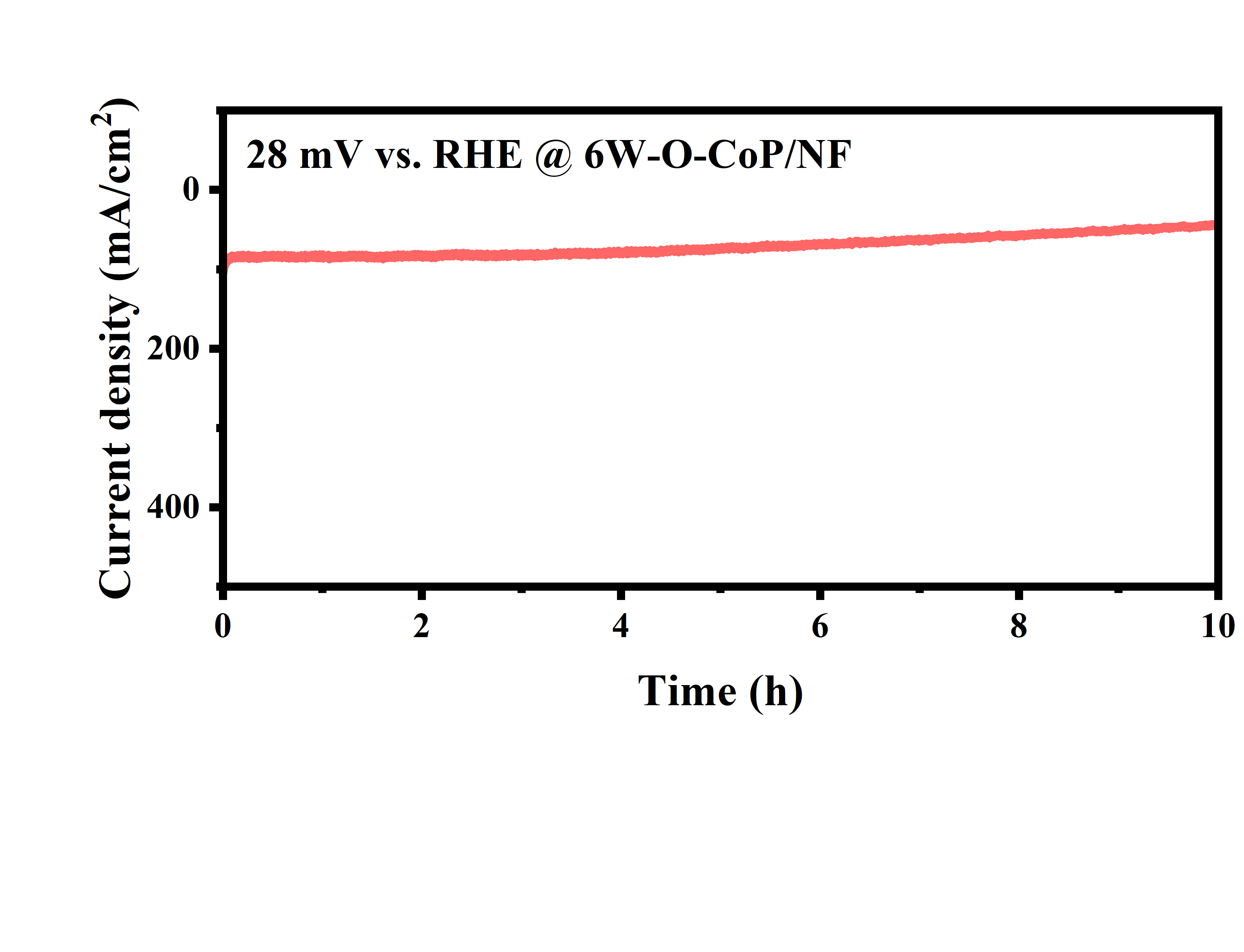


**Figure S29.** Stability measurement for 6W-O-CoP/NF at overpotential of 28 mV (vs. RHE) for 10 h.


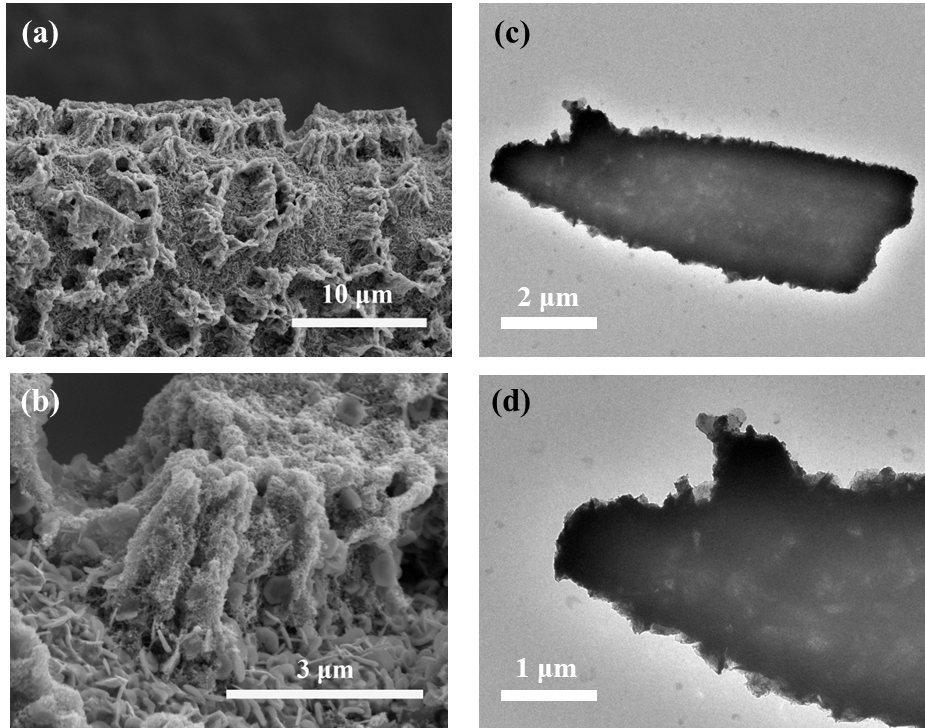


**Figure S30.** (a, b) SEM and (c, d) TEM images of the 6W-O-CoP/NF catalyst after 5000 cycles of HzOR test at different magnifications.


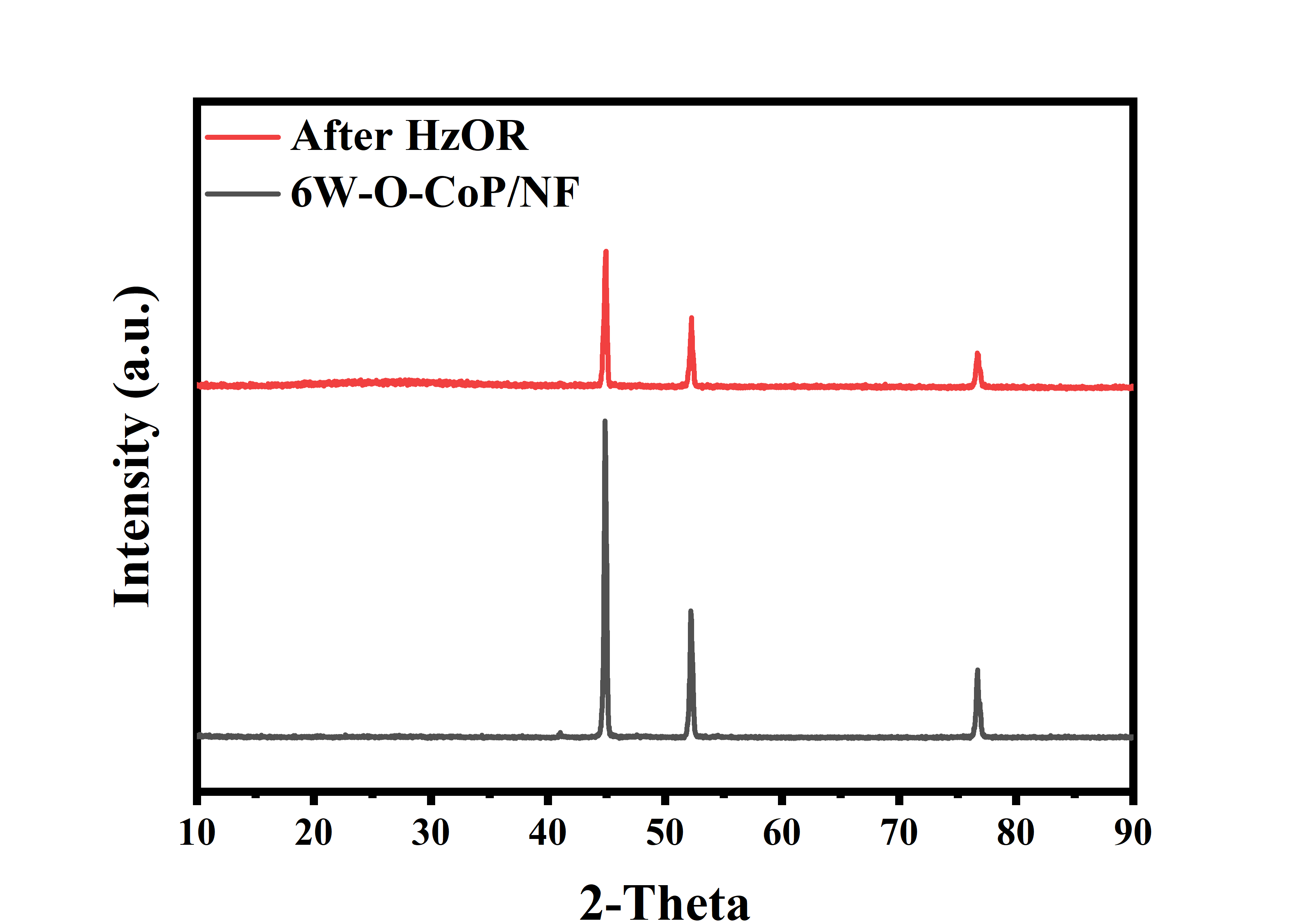


**Figure S31**. XRD patterns of 6W-O-CoP/NF catalyst before and after 5000 cycles of HzOR test.


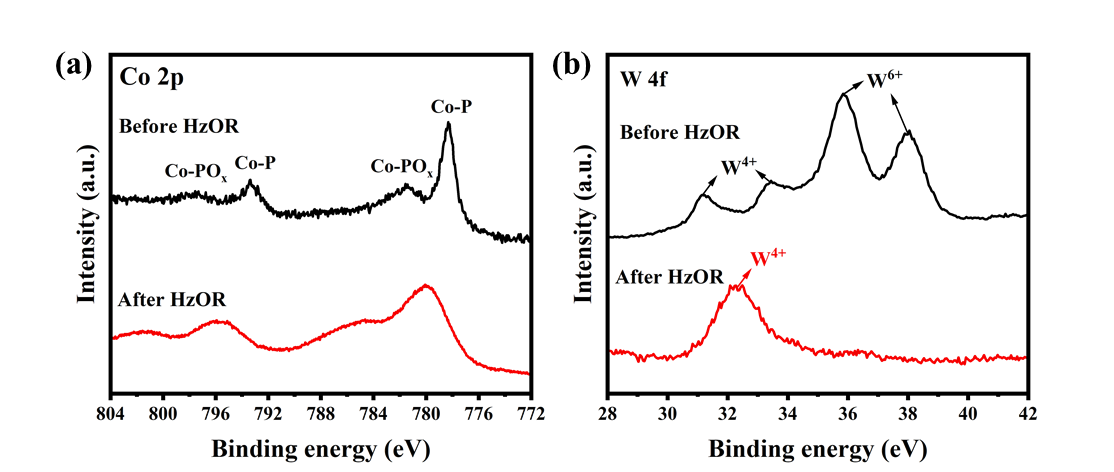


**Figure S32**. High resolution XPS spectra of (a) Co 2p and (b) W 4f for 6W-O-CoP/NF catalyst after 5000 cycles of HzOR test.

**
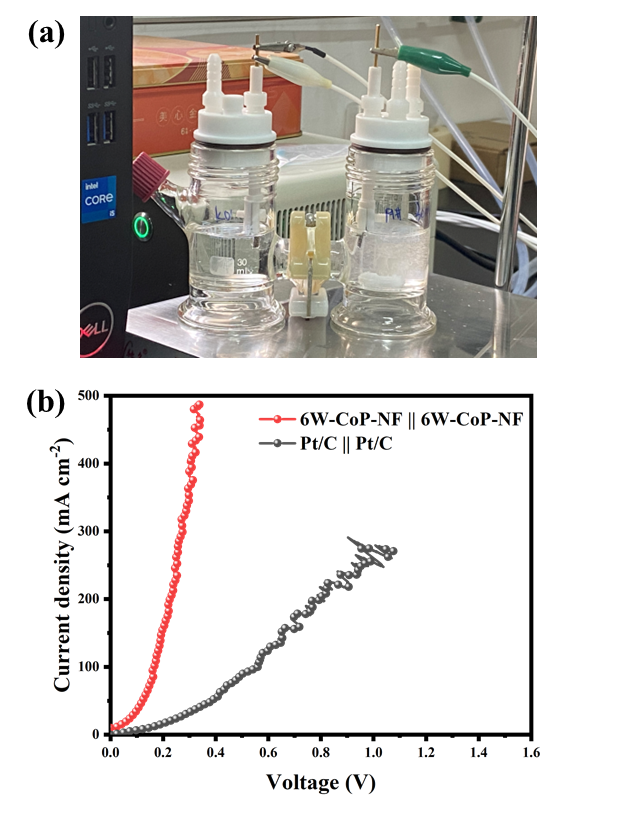
**

**Figure S33.** (a) The digital photo of assembled overall hydrazine splitting (OHzS) unit (6W-O-CoP/NF (+) || 6W-O-CoP/NF (-)). (b) LSV curves of OHzS electrolyzer equipped with 6W-O-CoP/NF (red) as both anode and cathode catalyst in 1.0 M KOH + 0.1M N_2_H_4_ electrolyte and that with commercial Pt/C as benchmarks measured for comparison (black).


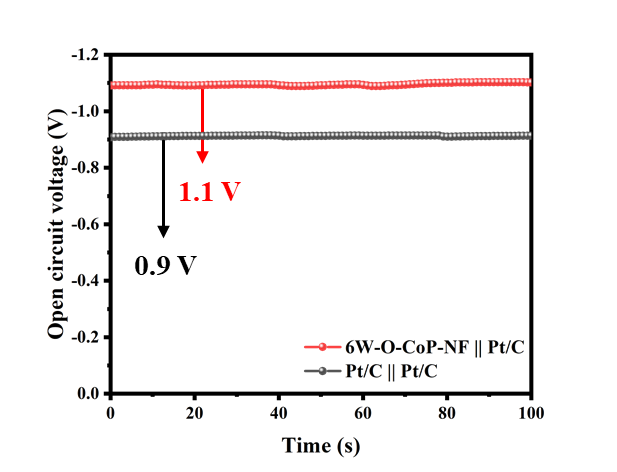


**Figure S34.** Open circuit voltages of 6W-O-CoP/NF || Pt/C and Pt/C || Pt/C.


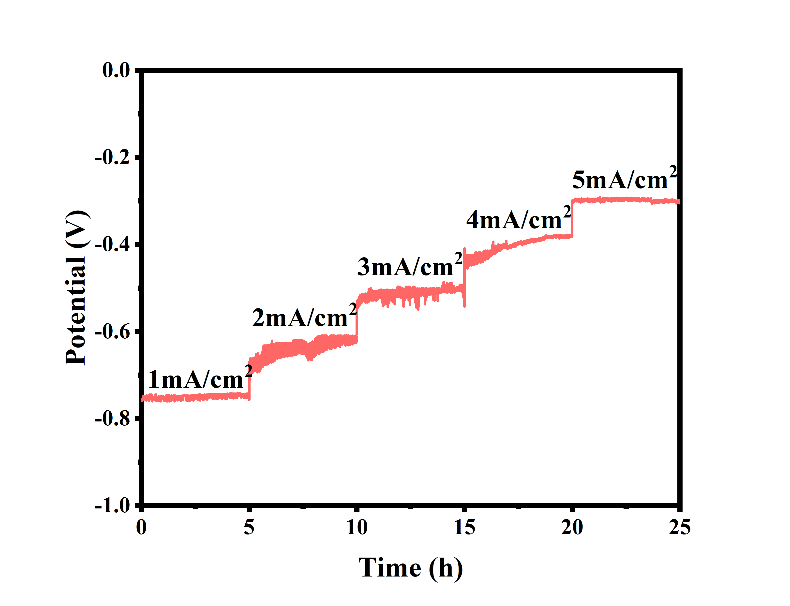


**Figure S35.** The stability measurement of assembled DHzFC at different current density.


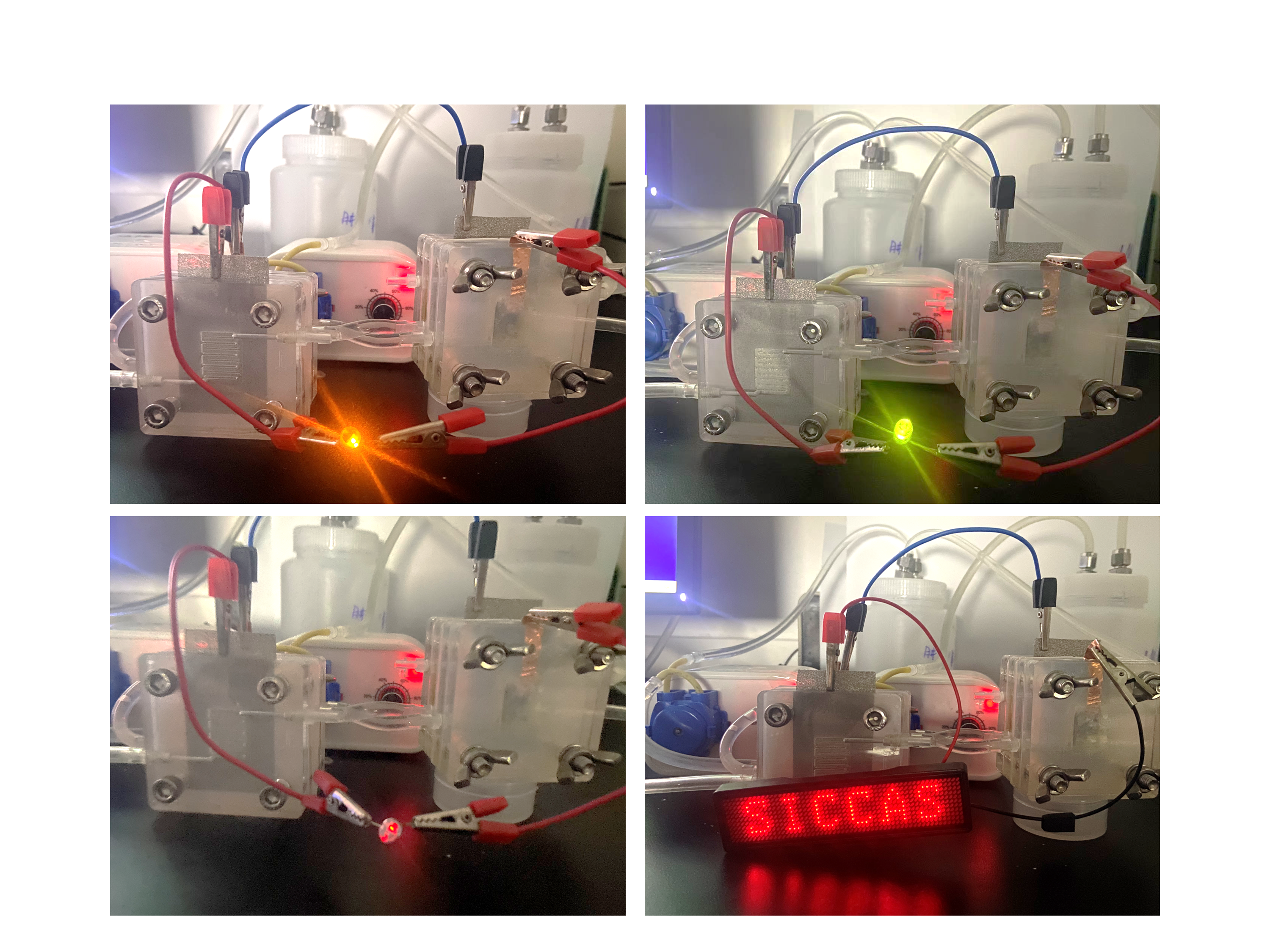


**Figure S36.** Digital photograph of different LEDs powered by the homemade DHzFC.

**Table S1.** Inductive Coupled Plasma (ICP) results of prepared samples.

| Samples | W  (wt.%) | Co  (wt.%) | P  (wt.%) |
| --- | --- | --- | --- |
| 0W-O-CoP/NF | 0.003 | 0.77 | 1.38 |
| 2W-O-CoP/NF | 0.93 | 0.77 | 0.87 |
| 4W-O-CoP/NF | 1.03 | 0.87 | 0.78 |
| 6W-O-CoP/NF | 1.39 | 0.97 | 1.06 |
| 10W-O-CoP/NF | 1.61 | 1.14 | 1.16 |

**Table S2.** Comparison of the HER performance in 1.0M KOH between 6W-O-CoP/NF in this work and various self-supported transition metal-based catalysts recently reported.

| Materials | Current density  (mA/cm^2^) | Potential  (V vs. RHE) | Reference |
| --- | --- | --- | --- |
| 6W-O-CoP/NF | 1000 | 185.6 | This work |
| A-NiCo LDH/NF | 1000 | 381 | ^1^ |
| Co-P Foam | 1000 | 290 | ^2^ |
| P-MNS/NF | 1000 | 245 | ^3^ |
| WMo-NiCoP/NF | 1000 | 249 | ^4^ |
| NiCoS@CoCH | 1000 | 438 | ^5^ |
| NiCo@C-NiCoMoO/NF | 1000 | 266 | ^6^ |
| H-CoS@NiFe LDH/NF | 1000 | 375 | ^7^ |
| N-NiMoS | 1000 | 322 | ^8^ |
| NiCoP-CPF | 1000 | 295 | ^9^ |
| Ru-CoO_x_/NF | 1000 | 252 | ^10^ |

**Table S3.** EIS parameters of obtained catalysts in 1.0M KOH toward HER.

| Materials | R (Ω) | R_1_ (Ω) | R_2_ (R_ct_, Ω) |
| --- | --- | --- | --- |
| P-NF | 1.67 | 35.33 | 14.66 |
| CoP/NF | 1.32 | 0.94 | 13.82 |
| 0W-O-CoP/NF | 1.27 | 0.81 | 6.29 |
| 2W-O-CoP/NF | 1.88 | 0.98 | 3.26 |
| 4W-O-CoP/NF | 1.77 | 0.88 | 2.54 |
| 6W-O-CoP/NF | 1.54 | 0.50 | 1.40 |
| 8W-O-CoP/NF | 1.55 | 0.40 | 4.64 |
| 10W-O-CoP/NF | 1.81 | 0.83 | 6.51 |

**Table S4.** Comparison of the HzOR performance between 6W-O-CoP/NF in this work and various catalysts recently reported.

| Materials | Current density  (mA/cm^2^) | Potential  (V vs. RHE) | Tafel slope  (mV/dec) | Reference |
| --- | --- | --- | --- | --- |
| 6W-O-CoP/NF | 100  200  300 | -0.035  -0.019  -0.005 | 8.43 | This work |
| Ru-MPNC | 10  200 | -0.039  0.072 | 40 | ^11^ |
| FeWO_4_/WO_3_ | 10 | -0.034 | 45.1 | ^12^ |
| NiCo-MoNi_4_ | 10 | -0.03 | - | ^13^ |
| Ni-C HNSA | 10 | -0.02 | 16.2 | ^14^ |
| Mo-Ni_3_N/NF | 10  200 | -0.0003  0.075 | 48 | ^15^ |
| FeNiP-NPHC | 100 | 0.007 | 24 | ^16^ |
| Ni-Co-Se | 300 | 0.4 | 88 | ^17^ |
| PtCu-NA | 200 | 0.668 | 40 | ^18^ |
| PW-Co_3_N/NC | 200 | 0.027 | 14 | ^19^ |

**Table S5.** EIS parameters of obtained catalysts in 1.0M KOH + 0.1M N_2_H_4_ toward HzOR.

| Materials | R (Ω) | R_1_ (Ω) | R_2_ (R_ct_, Ω) |
| --- | --- | --- | --- |
| 0W-O-CoP/NF | 1.46 | 0.98 | 1.53 |
| 2W-O-CoP/NF | 1.75 | 0.63 | 1.52 |
| 4W-O-CoP/NF | 2.50 | 0.12 | 1.32 |
| 6W-O-CoP/NF | 1.56 | 0.57 | 1.05 |
| 8W-O-CoP/NF | 1.53 | 0.71 | 1.35 |
| 10W-O-CoP/NF | 1.38 | 0.59 | 1.74 |

**Table S6.** Comparison of the DHzFC performance between 6W-O-CoP/NF in this work and various catalysts recently reported.

|  | Power density  (mW/cm^2^) | Open circuit voltage(V) | Reference |
| --- | --- | --- | --- |
| 6W-O-CoP/NF | 142 | 1.1 | This work |
| Ni_3_N-Co_3_N PNAs/NF | 60.3 | 1.00 | ^20^ |
| Mo Ni_3_N/Ni/NF | 37.8 | 0.96 | ^21^ |
| PW-Co_3_N NWA/NF | 46.3 | 0.98 | ^19^ |
| FeNiP-NPHC | 31 | 0.98 | ^16^ |
| Cu-CoFe/Co/NC | 26.7 | 0.996 | ^22^ |

1. **References**

1. H. Y. Yang, Z. L. Chen, P. F. Guo, B. Fei, R. B. Wu, B-doping-induced amorphization of LDH for large-current-density hydrogen evolution reaction. Appl. Catal. B: Environ. **261**, 118240 (2020). <https://doi.org/10.1016/j.apcatb.2019.118240>

2. Y. Li, B. Wei, Z. P. Yu, O. Bondarchuk, A. Araujo et al., Bifunctional Porous Cobalt Phosphide Foam for High-Current-Density Alkaline Water Electrolysis with 4000 h Long Stability. ACS Sustain. Chem. Eng. **8**, 10193-10200 (2020). <https://doi.org/10.1021/acssuschemeng.0c02671>

3. Y. Tong, D. M. Feng, P. Z. Chen, Dual Modification Strategy of Nickel Sulfide as pH-Universal Catalysts for Hydrogen Production at Large Current Density. ACS Sustain. Chem. Eng. **9**, 10601-10610 (2021). <https://doi.org/10.1021/acssuschemeng.1c02807>

4. X. Guo, M. G. Li, L. He, S. Geng, F. Y. Tian et al., Industrially promising NiCoP nanorod arrays tailored with trace W and Mo atoms for boosting large-current-density overall water splitting. Nanoscale **13**, 14179-14185 (2021). <https://doi.org/10.1039/D1NR03186D>

5. X. Zhang, R. J. Zheng, M. T. Jin, R. Shi, Z. Ai et al., NiCoS_x_@Cobalt Carbonate Hydroxide Obtained by Surface Sulfurization for Efficient and Stable Hydrogen Evolution at Large Current Densities. ACS Appl. Mater. Interfaces **13**, 35647-35656 (2021). <https://doi.org/10.1021/acsami.1c07504>

6. G. F. Qian, J. L. Chen, T. Q. Yu, L. Luo, S. B. Yin, N-Doped Graphene-Decorated NiCo Alloy Coupled with Mesoporous NiCoMoO Nano-sheet Heterojunction for Enhanced Water Electrolysis Activity at High Current Density. Nano-Micro Lett. **13**, 77 (2021). <https://doi.org/10.1007/s40820-021-00607-5>

7. Y. J. Lee, S. K. Park, Metal-Organic Framework-Derived Hollow CoS_x_ Nanoarray Coupled with NiFe Layered Double Hydroxides as Efficient Bifunctional Electrocatalyst for Overall Water Splitting. Small **18**, 2200586 (2022). <https://doi.org/10.1002/smll.202200586>

8. C. Q. Huang, L. Yu, W. Zhang, Q. Xiao, J. Q. Zhou et al., N-doped Ni-Mo based sulfides for high-efficiency and stable hydrogen evolution reaction. Appl. Catal. B: Environ. **276**, 119137 (2020). <https://doi.org/10.1016/j.apcatb.2020.119137>

9. X. G. Chen, X. Zhao, Y. Y. Wang, S. S. Wang, Y. Y. Shang et al., Layered Ni-Co-P Electrode Synthesized by CV Electrodeposition for Hydrogen Evolution at Large Currents. ChemCatChem **13**, 3619-3627 (2021). <https://doi.org/10.1002/cctc.202100707>

10. D. L. Wu, D. Chen, J. W. Zhu, S. C. Mu, Ultralow Ru Incorporated Amorphous Cobalt-Based Oxides for High-Current-Density Overall Water Splitting in Alkaline and Seawater Media. Small **17**, 2102777 (2021). <https://doi.org/10.1002/smll.202102777>

11. J. S. Wang, X. Y. Guan, H. B. Li, S. Y. Zeng, R. Li et al., Robust Ru-N metal-support interaction to promote self-powered H_2_ production assisted by hydrazine oxidation. Nano Energy **100**, 107467 (2022). <https://doi.org/10.1016/j.nanoen.2022.107467>

12. F. Shen, Z. L. Wang, Y. M. Wang, G. F. Qian, M. J. Pan et al., Highly active bifunctional catalyst: Constructing FeWO_4_-WO_3_ heterostructure for water and hydrazine oxidation at large current density. Nano Research **14**, 4356-4361 (2021). <https://doi.org/10.1007/s12274-021-3548-z>

13. Q. Z. Qian, Y. P. Li, Y. Liu, Y. M. Guo, Z. Y. Li et al., Hierarchical multi-component nanosheet array electrode with abundant NiCo/MoNi_4_ heterostructure interfaces enables superior bifunctionality towards hydrazine oxidation assisted energy-saving hydrogen generation. Chem. Eng. J. **414**, 128818 (2021). <https://doi.org/10.1016/j.cej.2021.128818>

14. Y. Zhu, J. H. Zhang, Q. Z. Qian, Y. P. Li, Z. Y. Li et al., Dual Nanoislands on Ni/C Hybrid Nanosheet Activate Superior Hydrazine Oxidation-Assisted High-Efficiency H_2_ Production. Angew. Chem. Int. Ed. **61**, e202113082 (2022). <https://doi.org/10.1002/anie.202113082>

15. Y. Liu, J. H. Zhang, Y. P. Li, Q. Z. Qian, Z. Y. Li et al., Realizing the Synergy of Interface Engineering and Chemical Substitution for Ni_3_N Enables its Bifunctionality Toward Hydrazine Oxidation Assisted Energy-Saving Hydrogen Production. Adv. Funct. Mater. **31** (2021). <https://doi.org/10.1002/adfm.202103673>

16. Q. P. Yu, X. B. Liu, G. S. Liu, X. P. Wang, Z. J. Li et al., Constructing Three-Phase Heterojunction with 1D/3D Hierarchical Structure as Efficient Trifunctional Electrocatalyst in Alkaline Seawater. Adv. Funct. Mater. **32**, 2205767 (2022). <https://doi.org/10.1002/adfm.202205767>

17. Z. B. Feng, E. P. Wang, S. Huang, J. M. Liu, A bifunctional nanoporous Ni-Co-Se electrocatalyst with a superaerophobic surface for water and hydrazine oxidation. Nanoscale **12**, 4426-4434 (2020). <https://doi.org/10.1039/C9NR09959J>

18. S. M. Ge, L. W. Zhang, J. R. Hou, S. Liu, Y. J. Qin et al., Cu_2_O-Derived PtCu Nanoalloy toward Energy-Efficient Hydrogen Production via Hydrazine Electrolysis under Large Current Density. ACS Appl. Energy Mater. **5**, 9487-9494 (2022). <https://doi.org/10.1021/acsaem.2c01006>

19. Y. Liu, J. H. Zhang, Y. P. Li, Q. Z. Qian, Z. Y. Li et al., Manipulating dehydrogenation kinetics through dual-doping Co_3_N electrode enables highly efficient hydrazine oxidation assisting self-powered H_2_ production. Nat. Commun. **11**, 1853 (2020). <https://doi.org/10.1038/s41467-020-15563-8>

20. Q. Z. Qian, J. H. Zhang, J. M. Li, Y. P. Li, X. Jin et al., Artificial Heterointerfaces Achieve Delicate Reaction Kinetics towards Hydrogen Evolution and Hydrazine Oxidation Catalysis. Angew. Chem. Int. Ed. **60**, 5984-5993 (2021). <https://doi.org/10.1002/anie.202014362>

21. Y. Liu, J. H. Zhang, Y. P. Li, Q. Z. Qian, Z. Y. Li, G. Q. Zhang, Realizing the Synergy of Interface Engineering and Chemical Substitution for Ni_3_N Enables its Bifunctionality Toward Hydrazine Oxidation Assisted Energy-Saving Hydrogen Production. Adv. Funct. Mater. **31**, 2103673 (2021). <https://doi.org/10.1002/adfm.202103673>

22. X. B. Liu, H. M. Mao, G. S. Liu, Q. P. Yu, S. Q. Wu et al., Metal doping and Hetero-engineering of Cu-doped CoFe/Co embedded in N-doped carbon for improving trifunctional electrocatalytic activity in alkaline seawater. Chem. Eng. J. **451**, 138699 (2023). <https://doi.org/10.1016/j.cej.2022.138699>
